# Distinct interactions of Sox5 and Sox10 in fate specification of pigment cells in medaka and zebrafish

**DOI:** 10.1371/journal.pgen.1007260

**Published:** 2018-04-05

**Authors:** Yusuke Nagao, Hiroyuki Takada, Motohiro Miyadai, Tomoko Adachi, Ryoko Seki, Yasuhiro Kamei, Ikuyo Hara, Yoshihito Taniguchi, Kiyoshi Naruse, Masahiko Hibi, Robert N. Kelsh, Hisashi Hashimoto

**Affiliations:** 1 Bioscience and Biotechnology Center and Division of Biological Science, Graduate School of Science, Nagoya University, Furo-cho, Chikusa-ku, Nagoya, Aichi, Japan; 2 Department of Biology and Biochemistry, University of Bath, Claverton Down, Bath, United Kingdom; 3 Department of Basic Biology, School of Life Science, Graduate University of Advanced Studies (SOKENDAI), Myodaiji, Okazaki, Aichi, Japan; 4 Spectrography and Bioimaging Facility, National Institute for Basic Biology, Myodaiji, Okazaki, Aichi, Japan; 5 Laboratory of Bioresources, National Institute for Basic Biology, Myodaiji, Okazaki, Aichi, Japan; 6 Department of Public Health and Preventive Medicine, Kyorin University, School of Medicine, Mitaka, Tokyo, Japan; University of Wuerzburg, GERMANY

## Abstract

Mechanisms generating diverse cell types from multipotent progenitors are fundamental for normal development. Pigment cells are derived from multipotent neural crest cells and their diversity in teleosts provides an excellent model for studying mechanisms controlling fate specification of distinct cell types. Zebrafish have three types of pigment cells (melanocytes, iridophores and xanthophores) while medaka have four (three shared with zebrafish, plus leucophores), raising questions about how conserved mechanisms of fate specification of each pigment cell type are in these fish. We have previously shown that the Sry-related transcription factor Sox10 is crucial for fate specification of pigment cells in zebrafish, and that Sox5 promotes xanthophores and represses leucophores in a shared xanthophore/leucophore progenitor in medaka. Employing TILLING, TALEN and CRISPR/Cas9 technologies, we generated medaka and zebrafish *sox5* and *sox10* mutants and conducted comparative analyses of their compound mutant phenotypes. We show that specification of all pigment cells, except leucophores, is dependent on Sox10. Loss of Sox5 in Sox10-defective fish partially rescued the formation of all pigment cells in zebrafish, and melanocytes and iridophores in medaka, suggesting that Sox5 represses Sox10-dependent formation of these pigment cells, similar to their interaction in mammalian melanocyte specification. In contrast, in medaka, loss of Sox10 acts cooperatively with Sox5, enhancing both xanthophore reduction and leucophore increase in *sox5* mutants. Misexpression of Sox5 in the xanthophore/leucophore progenitors increased xanthophores and reduced leucophores in medaka. Thus, the mode of Sox5 function in xanthophore specification differs between medaka (promoting) and zebrafish (repressing), which is also the case in adult fish. Our findings reveal surprising diversity in even the mode of the interactions between Sox5 and Sox10 governing specification of pigment cell types in medaka and zebrafish, and suggest that this is related to the evolution of a fourth pigment cell type.

## Author summary

How individual cell fates become specified from multipotent progenitors is a fundamental question in developmental and stem cell biology. Body pigment cells derive from a multipotent progenitor, but while in zebrafish there are three types of pigment cells (melanocytes, iridophores and xanthophores), in medaka these progenitors form four (as zebrafish, plus leucophores). Here, we address whether mechanisms generating each cell-type are conserved between the two species. We focus on two key regulatory proteins, Sox5 and Sox10, which we previously showed were involved in pigment cell development in medaka and zebrafish, respectively. We compare experimentally how the two proteins interact in regulating development of each of the pigment cell lineages in these fish. We show that development of all pigment cells, except leucophores, is dependent on Sox10, and that Sox5 modulates Sox10 activity antagonistically in all pigment cells in zebrafish, and melanocytes and iridophores in medaka. Surprisingly, in medaka, Sox5 acts co-operatively with Sox10 to promote xanthophore fate and to repress leucophore fate. Our findings reveal surprising diversity how Sox5 and Sox10 interact to govern pigment cell development in medaka and zebrafish, and suggest that this likely relates to the evolution of the novel leucophore pigment cell type in medaka.

## Introduction

Cell fate specification from multipotent stem cells is a fundamental process during embryonic development. The mechanisms determining how multipotent progenitors choose a diversity of different cell fates is key to understanding the generation of multiple cell types in the animal body. The neural crest is a population of migratory multipotent cells that form at the boundary of neural plate and non-neural ectoderm, and gives rise to a variety of cell types including pigment cells [1, 2]. Whereas mammals and birds have a single type of pigment cells, the black or brown melanocyte, fish have multiple types of pigment cells, known as chromatophores [3, 4]. In zebrafish there are three types–melanophores (often referred to as melanocytes, the term we shall use throughout this manuscript hereafter), iridescent iridophores, and yellow/orange xanthophores [5]. Medaka has four chromatophore types, with all those from zebrafish plus reflective white (or orange during embryonic/larval stages) leucophores [6–8], raising the question of whether fate specification mechanisms remain conserved through evolution. Thus, the multitude of pigment cell types within fish makes pigment cells an excellent ‘model within a model’ system for studying fate specification and its evolution, especially since they are both easily identified by their natural colors and other markers, and not essential for survival [4].

The similarity of embryonic/larval pigment pattern in medaka and zebrafish, species that last shared a common ancestor some 324 million years ago [9], makes these two fish models highly complementary and valuable for comparative studies of neural crest. Melanocytes form three longitudinal stripes, known as the dorsal stripe (DS), lateral stripe (LS) and ventral stripe (VS) in both zebrafish and medaka, and a fourth stripe on the ventral yolk sac, called the yolk sac stripe in zebrafish or yolk sac cluster (YSC) in medaka; xanthophores lie under the epidermis between the stripes in both species; iridophores locate on the dorsal yolk sac in clusters, called lateral patches (LP), as well as on the eyes in both species. Iridophores are distributed along the dorsal midline in zebrafish, being surrounded by melanocytes, whereas leucophores occupy this position in medaka [5, 8]. Whereas melanocytes, iridophores and xanthophores are widely distributed in teleosts [4], leucophores have a much more restricted distribution and hence are likely an evolutionary innovation in the medaka lineage [10–13].

It has been proposed that all types of pigment cells are generated from common pigment cell progenitors (chromatoblasts) [14] via progressive fate-restriction processes [15, 16]. Leucophores are present in medaka but not in zebrafish, implying an additional choice between alternative fates in medaka. Our previous studies showed a close developmental and genetic relationship between leucophores and xanthophores [17]. This led us to propose that both leucophore and xanthophore specification from the neural crest is Pax7a-dependent and that they may develop from a shared bipotent progenitor, perhaps distinguishable by its expression of *pax7a*. The Sry-related high-mobility group (HMG) transcription factor Sox5 is widely expressed in migrating neural crest cells, but functions cell-autonomously in the *pax7a*-expressing shared progenitors to promote a xanthophore fate choice over leucophore [18].

Genetic analyses have identified other key transcription factors involved in pigment cell specification. The best studied of these is Mitf (microphthalmia-associated transcription factor; Mitfa in zebrafish), which is considered the master regulator of melanocyte development in zebrafish and mammals [19–21]. Mitf controls expression of genes required for melanocyte development, including *dopachrome tautomerase* (*dct*), *tyrosinase* and the receptor tyrosine kinase *c-kit* [22]. Zebrafish *mitfa* mutants show reduced or no melanocytes, but an increased number of iridophores [19]. Expression of *Mitf/mitfa* is regulated by Sox10, which is widely expressed in neural crest cells prior to their differentiation [23–28]. As loss of Sox10 function results in complete or partial absence of all pigment cells and most other neural-crest derivative cells in zebrafish and mouse, Sox10 is necessary for specification of not only the melanocyte lineage, but also for all non-ectomesenchymal lineages, including xanthophores and iridophores (e.g.[23, 29–35] and reviewed in [23]). Although the role of *sox10* has never been explored in medaka, we predict a key role in specification of the three pigment cell types shared with zebrafish; whether that role extends to leucophores remains to be tested.

Among the Sox-family transcription factors [36], the SoxD subgroup (Sox5, Sox6 and Sox13), is unique in lacking any known trans-activation or -repression domains; thus, they exert their functions via obligatory interactions with other transcription factors [37]. The SoxE subgroup (Sox8, Sox9 and Sox10) are key partners for these SoxD factors (reviewed in [37]). In mammalian chondrocyte formation, neither Sox5/6 nor Sox9 alone efficiently elicits expression of chondrocyte-specific genes, but together they function synergistically in their transcription [38, 39]. In contrast, in mammalian oligodendrocytes and melanoblasts, Sox5 and/or Sox6 counteract transcriptional activation by Sox9 or Sox10 [40, 41]. In mouse, Sox5 is dispensable for melanocyte development, but its loss in *Sox10* heterozygotes partially rescues a severe reduction of melanoblasts and expression of *Mitf* and *Dct* [41]. Although Sox5 and Sox10 are involved in pigment cell development in fish, it remains unclear whether Sox5 and Sox10 interact in the specification of multiple types of pigment cells in fish. Furthermore, the extent of species-specific variation in these interactions and roles within the highly diverse fish has never been explored.

In this study we have compared pigment cell phenotypes of single and compound medaka and zebrafish *sox5* and *sox10* mutants, and *sox5*-misexpressing medaka. We demonstrate that Sox5 acts antagonistically against Sox10 in the specification of all pigment cell lineages in zebrafish and in melanocyte and iridophore lineages in medaka, whereas synergistic interactions between Sox5 and Sox10 play important roles in specification of xanthophore and leucophore lineages in medaka. Our findings indicate that there are distinct interactions between Sox5 and Sox10 that contribute to generation of different types of pigment cells, and that many, but surprisingly not all, of these are conserved between zebrafish and medaka. Intriguingly, the changed mode of interactions between Sox5 and Sox10 affect the xanthophore, and correlate with the evolution of a novel pigment cell type (leucophore); given the shared developmental origin of leucophores and xanthophores in medaka, we hypothesize that the switch in Sox5-Sox10 interactions reflects the evolution of this novelty.

## Results

### Medaka *sox10a* and *sox10b* are expressed in neural crest cells

**Fig 1:**
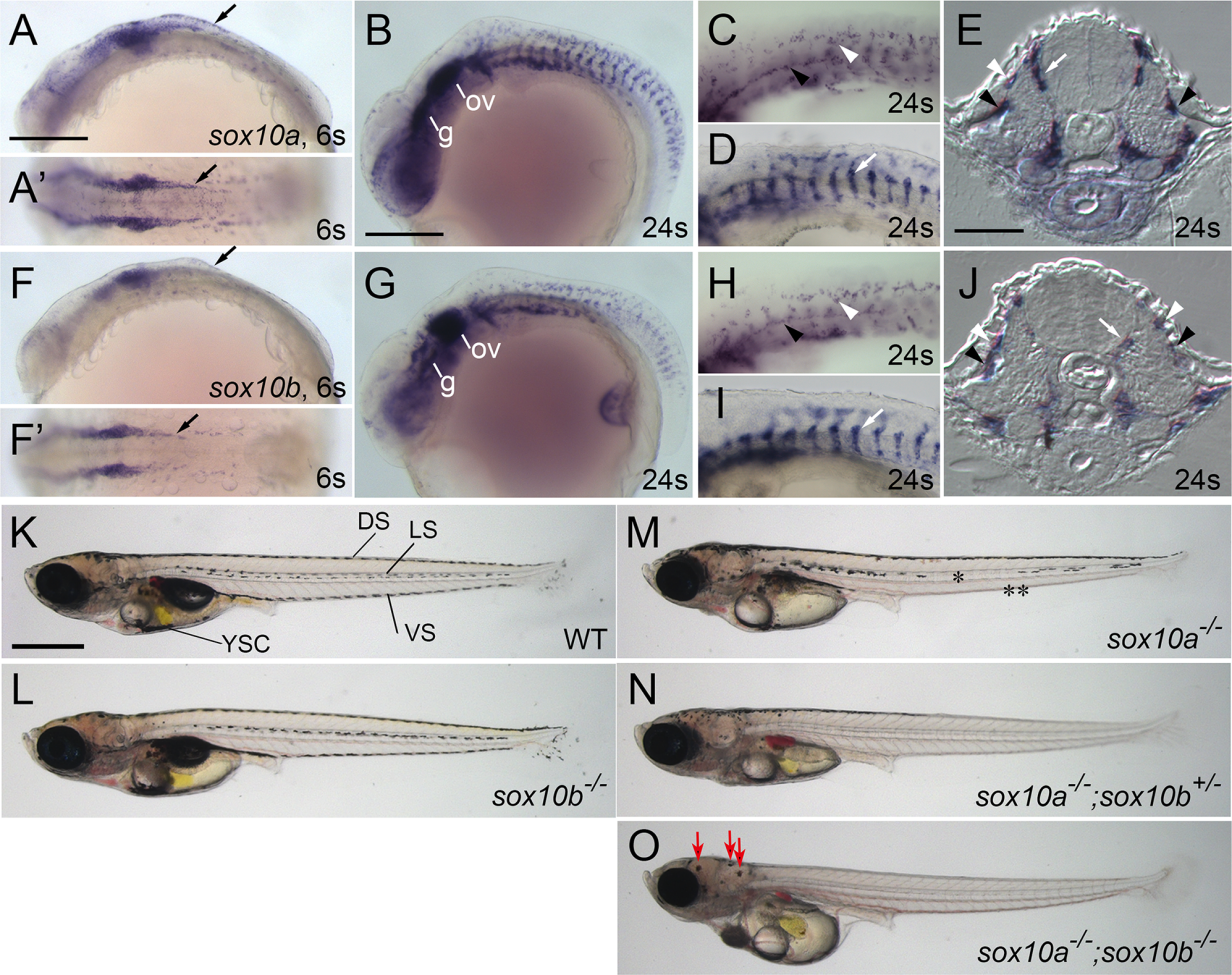
**Medaka *sox10a* and *sox10b* are expressed in neural crest and their loss of function affects pigment cell development**. (A-E) *sox10a*. (F-J) *sox10b*. (E, J) Transverse sections. (A, B, C, D, F, G, H, I) Lateral views. (A’, F’) Dorsal views. (K-O) 9 dpf. Lateral views. (A, F) At 6-somite stage (6s, 34 hpf), both *sox10a* and *sox10b* are expressed in premigratory neural crest cells (black arrows). (B-E, G-J) At 24-somite stage (24s, 58 hpf), *sox10a-* and *sox10b-* expressing cells are found in glial precursors on the posterior lateral line nerve (black arrowheads in C, E, H and J) and migrating neural crest cells between neural tube and somite (medial pathway, white arrows in D, E, I and J) and between epidermis and somite (lateral pathway, white arrowheads in C, E, H and J). (B, G) In head, both *sox10a* and *sox10b* are expressed in otic vesicle (ov) and cranial ganglia (g). (C, H) Close-up images of lateral trunk surface. (D, I) Close-up images of migrating neural crest through medial pathway. (K, L) WT and *sox10b*^*-/-*^ hatchlings show a normal pigment pattern, composed of dorsal stripe (DS), lateral stripe (LS), ventral stripe (VS) and yolk sac cluster (YSC). (M) In *sox10a*^*-/-*^ mutants, melanocytes in LS (*) and in posterior side of VS (from caudal end of yolk sac to tail, **) are severely reduced. (N) *sox10a*^*-/-*^*;sox10b*^*+/-*^ mutants retain melanocytes only in the head and the anterior part of the body. (O) *sox10a*^*-/-*^*;sox10b*^*-/-*^ mutants lack all chromatophores, except that leucophores on the head are retained (red arrows, see also Fig 4D). Scale Bars: (A) 200 μm, also applied to A’, F and F’; (B) 200 μm, also applied to G; (E) 20 μm, also applied to J; (K) 250μm, also applied to L, M, N and O.

Although major vertebrate species, such as mouse and zebrafish, have only one *sox10* gene, some fish species including medaka have two *sox10* genes, *sox10a* and *sox10b* [42]. In medaka, like in other vertebrates, [30, 32, 33, 35, 43–45], expression of both *sox10a* and *sox10b* was detected in neural crest cells, both prior to and during migration, and in otic vesicle (Fig 1A–1J). At 6-somite stage (34 hpf), expression was detected in premigratory neural crest cells in the head and anterior trunk (Fig 1A, 1A’, 1F and 1F’), and then gradually extended posteriorly and ventrally through the trunk (S1A and S1B Fig). At 24-somite stage (58 hpf), *sox10a-* or *sox10b-*expressing neural crest cells were observed on both the medial and lateral pathway and in lines of cells that from their position in the horizontal myoseptum and their organization we identify as Schwann cell precursors associated with the posterior lateral line nerve (Fig 1B–1E and 1G–1J). This in situ analysis suggested that in medaka *sox10a* and *sox10b* were expressed in neural crest cells in a pattern similar to *sox5* [18]. To examine the degree of overlap of expression of medaka *sox10* genes with *sox5*, we used double whole mount in situ hybridization using Tyramide signal amplification (TSA) (S1C–S1E Fig). We note the extensive overlap between *sox10a* and *sox5* in numerous migrating neural crest cells (S1C and S1D Fig) and also in premigratory neural crest, which possess high multipotency [46] (S1C and S1E Fig). These observations of overlapping *sox10* and *sox5* expression lead us to examine if Sox5 and Sox10 function together in pigment cell development.

### Engineered mutant alleles for medaka *sox10a* and *sox10b* show defects in pigment cell development

We engineered medaka mutants for *sox10a* and *sox10b* using TALEN [47] and TILLING methods [48, 49]. By a TALEN, we established two *sox10a* mutant lines (*sox10a*^*E1ins10*^ and *sox10a*^*E2del16*^). The *sox10a*^*E2del16*^ allele has a 16-base nucleotide deletion in the second exon and so is predicted to generate a truncated Sox10a protein lacking the C-terminal of HMG DNA binding domain and the transactivation domain; the *sox10a*^*E1ins10*^ allele is predicted to give a similar, but even more severely truncated protein (S2 Fig, see the legend of S2 Fig for information of *sox10a*^*E1ins10*^ allele). Similarly, we obtained the *sox10b*^*E1del7*^ mutant allele, which has a 7-base nucleotide deletion in the first exon resulting in lack of most functional domains. From a medaka TILLING library, we isolated two *sox10b* mutant alleles, *sox10b*^*N108S*^ and *sox10b*^*F110L*^. They each have a distinct single nucleotide mutation which results in an amino acid substitution of a highly conserved amino acid in the HMG domain of Sox10b (*sox10b*^*N108S*^: N108S and *sox10b*^*F110L*^: F110L, S2 Fig). In the following study, we used the *sox10a*^*E2del16*^ and *sox10b*^*N108S*^ alleles unless specified, since we found no difference in phenotypes between E1ins10 and E2del16 alleles, nor among the N108S, F110L and E1del7 alleles.

We began by gross characterization of the pigment cell phenotypes of these *sox10* mutants (Fig 1K–1O). The *sox10a* homozygotes showed reduction of the number of pigment cells, but remaining cells were of normal morphology and pigmentation. They had severe defects in melanocyte numbers in the posterior and ventral body, reduced leucophores in the posterior body, and reduction of xanthophores (Fig 1M). The *sox10b* mutation(s) did not show any overt pigment cell phenotype (Fig 1L). In the compound *sox10a* homozygote and *sox10b* heterozygote mutants, the pigment cell phenotypes were more severe than those of *sox10a* homozygotes: all pigment cell types were strongly reduced in numbers except melanocytes and leucophores in the anterior body (Fig 1N). The phenotypes of the double homozygous *sox10a*;*sox10b* mutants were strikingly reminiscent of that of zebrafish *colourless/sox10* mutants [29, 30], lacking all pigment cells, although notably leucophores remained present on the dorsal head (Fig 1O). We note that in every case where a pigment cell phenotype was seen, it was restricted to the number of each pigment cell type–remaining cells looked normal in morphology and pigmentation. Thus, both Sox10a and Sox10b are required for development of pigment cells, except for leucophores on the head, and they have partially redundant functions in medaka.

### Sox5 counteracts Sox10 in fate specification of the melanocyte and iridophore lineages

**Fig 2:**
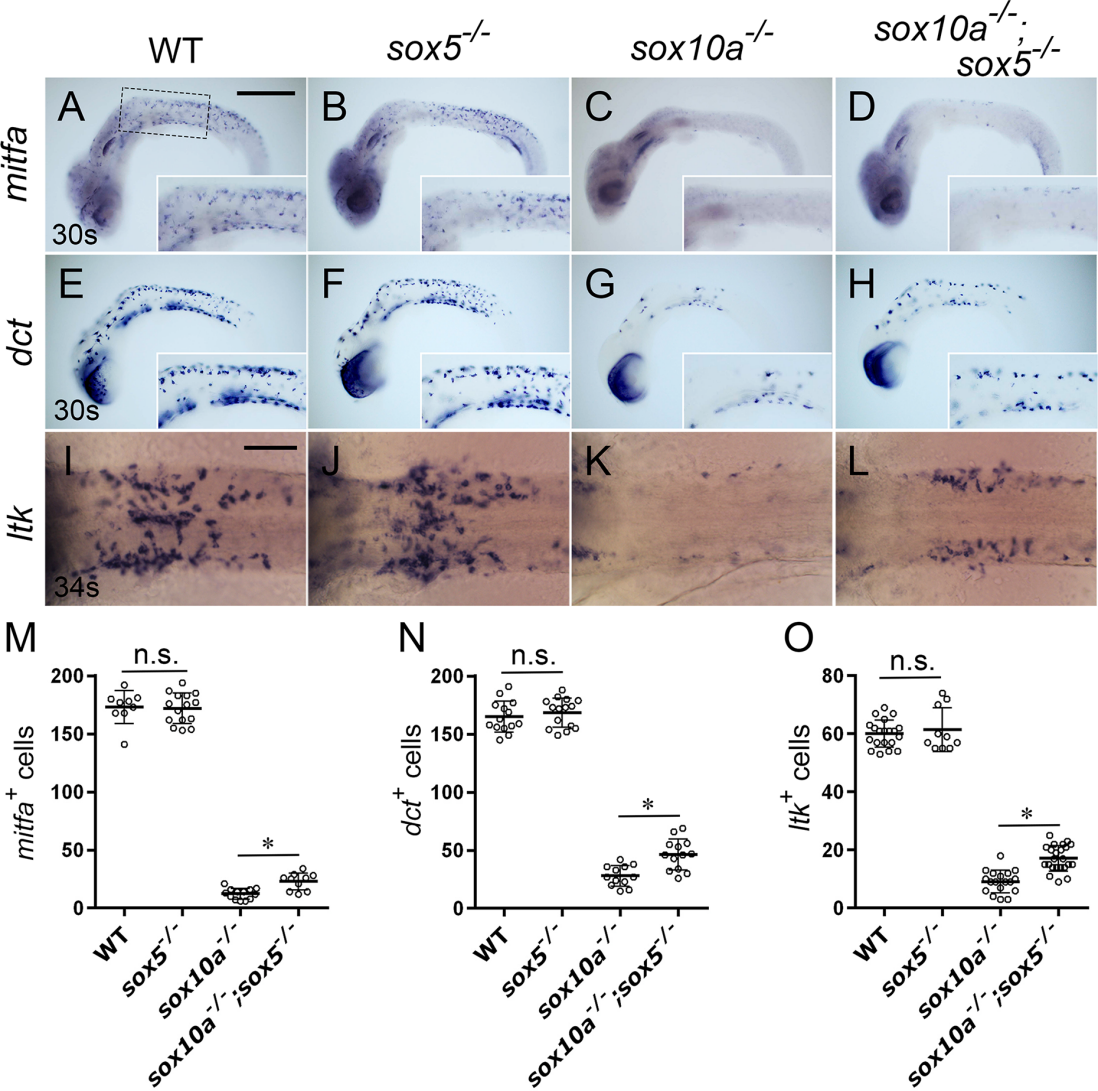
**Reductions in melanoblast and iridoblast specification in *sox10a* mutants were partially rescued by loss of *sox5***. (A-D) *mitfa*. (E-H) *dct*. (I-L) *ltk*. (A, E, I) WT. (B, F, J) *sox5*^*-/-*^. (C, G, K) *sox10a*^*-/-*^. (D, H, L) *sox10a*^*-/-*^*;sox5*^*-/-*^. (M-O) Quantitation of number of cells expressing each fate marker in the whole embryonic body (M, N) and in the lateral patches (O). (A-H) Lateral views. (I-L) Dorsal views. (A-H) 30-somite stage (30s, 64 hpf). (I-L) 34-somite stage (34s, 74 hpf). The box in (A) indicates the enlarged region (anterior trunk) in insets. Melanoblasts, defined by expression of *mitfa* and *dct*, are unaltered in the *sox5*^-/-^ mutant (B, F) as compared with WT (A, E). Expression of *mitfa* and *dct* are both dramatically reduced in *sox10a*^*-/-*^ mutants (C, G), but substantially recovered in *sox10a*^*-/-*^*;sox5*^*-/-*^ mutants (D, H). The number of *mitfa* (M, *mitfa*^*+*^)- or *dct* (N, *dct*^*+*^)- expressing cells is not different between WT (*mitfa*, n = 9; *dct*, n = 14) and *sox5*^*-/-*^ mutants (*mitfa*, n = 15; *dct*, n = 15). *p-*values are *p* = 0.78 (*mitfa*) and *p* = 0.52 (*dct*). *sox10a*^*-/-*^ mutants have significantly fewer of those cells (*mitfa*, n = 13; *dct*, n = 12) than WT (*p*<0.05 (m*itfa*) and *p*<0.05 (*dct*)). In the *sox10a*^*-/-*^*;sox5*^*-/-*^ double mutants (*mitfa*, n = 10; *dct*, n = 13), cell counts are significantly increased as compared with *sox10a*^*-/-*^ single mutant (*, *p*<0.05). *p*-values were calculated by Mann-Whitney test. Iridoblasts, as evidenced by *ltk* expression, in WT (I), *sox5*^*-/-*^ mutant (J), *sox10a*^*-/-*^ mutant (K) and *sox10a*^*-/-*^*;sox5*^*-/-*^ mutant (L) are shown for the lateral patches, a region of concentrated iridophores dorsolateral to the developing gut in the anterior trunk. The number of *ltk-*expressing cells (O, *ltk*^*+*^) is indistinguishable between WT (I, n = 20) and the *sox5*^*-/-*^ mutant (J, n = 10) (*p =* 0.98). In the *sox10a*^*-/-*^ mutant (K, n = 18), the number is significantly decreased as compared with WT (I). Again, iridoblasts cell counts between the *sox10a*^*-/-*^ mutant and the *sox10a*^*-/-*^*;sox5*^*-/-*^ mutant (L, n = 23) is statistically different (*, *p*<0.05), showing partial rescue in the double homozygote. *p*-values were calculated by Mann-Whitney test. (M-O) Bars show mean and error bar (s.d.). Scale bars: (A) 200 μm, (I) 40 μm.

To ask if Sox10a and Sox10b function with Sox5 in the pigment cell development in medaka, we generated compound mutants of *sox5*^*ml-3*^ and *sox10a*^*E2del16*^ and/or *sox10b*^*N108S*^. We refer to *sox5*^*ml-3*^ homozygotes, *sox10a*^*E2del16*^ homozygotes and *sox10b*^*N108S*^ homozygotes as *sox5*^*-/-*^, *sox10a*^*-/-*^ and *sox10b*^*-/-*^, respectively. We first examined the expression of the melanoblast markers *mitfa* and *dct* to elucidate the roles of *sox5*, *sox10a* and *sox10b* in melanocyte lineage specification (Fig 2A–2H, 2M and 2N). As described previously [18], numbers of *mitfa-* or *dct-*expressing cells were comparable between *sox5*^*-/-*^ and wild-type (WT) embryos, suggesting that *sox5* mutation has little effect on melanoblast formation (Fig 2B, 2F, 2M and 2N). In *sox10a*^*-/-*^ embryos, significant decrease of *mitfa-*expressing and *dct*-expressing cells was observed (Fig 2C, 2G, 2M and 2N), indicating a key role for Sox10a in medaka melanocyte fate specification. *sox10a*^*-/-*^*;sox5*^*-/-*^ double mutant embryos had fewer *mitfa-*expressing and *dct-*expressing cells than WT. However the numbers of those cells were larger in *sox10a*^*-/-*^*;sox5*^*-/-*^ double mutant embryos than in *sox10a*^*-/-*^ mutant (Fig 2D, 2H, 2M and 2N), indicating that loss of Sox5 led to partial rescue of the melanoblast specification defect in the *sox10a*^*-/-*^ embryos.

We next examined iridophore specification in the mutants using *leukocyte tyrosine kinase (ltk)* as an early iridophore marker, which is known to be expressed in multipotent neural crest cells at early stages and later specifically in the iridophore lineage in zebrafish [46]. In medaka, *ltk* expression was detected in premigratory neural crest cells in the cranial and anterior trunk neural crest at 12-somite stage (41 hpf, S3A Fig). At later stages, *ltk*-expressing cells appeared on the eyes (S3B Fig) and in the LP on the dorsal yolk sac [8] (S3C and S3C’ Fig). The position and density of these cells are consistent with those of differentiating iridoblasts. Numbers of the *ltk*-expressing cells on the yolk sac at 34-somite stage (74 hpf) in *sox5*^*-/-*^ embryos were indistinguishable from that in WT (Fig 2I, 2J and 2O). In *sox10a*^*-/-*^ embryos, significant reduction of the *ltk*-expressing cells was observed (Fig 2K and 2O), but it was significantly rescued in *sox10a*^*-/-*^*;sox5*^*-/-*^ double mutants (Fig 2L and 2O). Our results indicate that Sox5 functions antagonistically against Sox10 in specification of both melanocyte and iridophore lineages in medaka.

### Interaction of Sox5 and Sox10 influences late development of melanocytes and iridophores

We then examined the long-term consequences of loss-of-function of Sox5 and Sox10 on development of pigment cells in hatchlings (9 dpf, S4 Fig). Both *sox10a*^*-/-*^*;sox10b*^*-/-*^ and *sox10a*^*-/-*^*;sox10b*^*-/-*^*;sox5*^*-/-*^ mutants displayed complete loss of melanocytes (S4D and S4H Fig), indicating that Sox10 is absolutely required for melanocyte specification, even in the absence of Sox5. Conversely, *sox10b*^*-/-*^*;sox5*^*-/-*^ double mutants were not significantly different to *sox10b*^*-/-*^ mutants, being indistinguishable from WT siblings (S4X Fig), and showing that *sox10b* is insufficient to numerically affect melanocyte numbers, even in the absence of *sox5*. Despite the severe decrease in the cells expressing the melanoblast markers at early stages (Fig 2M and 2N), we found a slight increase in the total number of melanocytes in *sox10a*^*-/-*^ mutant larvae compared to WT siblings (S4X Fig). In contrast, loss of *sox5* activity in the context of either *sox10a*^*-/-*^ or *sox10a*^*-/-*^;*sox10b*^*+/-*^ mutants resulted in partial rescue of the melanocyte phenotype (S4F, S4G and S4X Fig), suggesting that the increased *dct*-expressing melanoblasts gave rise to increased melanocytes later in the double mutant, and that Sox5 acts to repress Sox10-dependent melanocyte formation.

In the course of these studies we also noted interesting, but complex, region-specific differences in the effects of these mutations. In medaka *sox10a*^*-/-*^ mutants, melanocytes were preferentially decreased in VS posterior to the caudal end of yolk sac (posterior VS) compared to DS, and in the LS (S4B, S4U and S4V Fig), as is reported for zebrafish hypomorphic *sox10* alleles [5, 50]. Unexpectedly, quantitative assessment revealed that *sox10a*^*-/-*^ mutants showed an increase in head melanocytes (S4S Fig), and in the DS and anterior VS (S4T and S4W Fig). In *sox10a*^*-/-*^*;sox10b*^*+/-*^ mutants, melanocytes were almost completely absent from the LS and the posterior VS (S4C, S4U and S4V Fig), but with variable, but often rather few residual melanocytes located in the DS, on the head and in the anterior VS (S4S, S4T, S4W and S4X Fig).

Both *sox10a*^*-/-*^*;sox10b*^*-/-*^ and *sox10a*^*-/-*^*;sox10b*^*-/-*^*;sox5*^*-/-*^ mutant larvae had no iridophores on LP (S4M and S4R Fig), indicating that Sox10 is absolutely required for specification of iridophores, even in the absence of Sox5. Although *sox10a*^*-/-*^*;sox10b*^*+/-*^ mutant showed strong reduction of iridophores, loss of *sox5* rescued iridophores in hatchlings (S4L and S4Q Fig), suggesting Sox5 also represses Sox10-dependent specification of iridophores.

In summary, Sox10 function is absolutely required for specification of melanocyte and iridophore lineages in medaka, and Sox5 antagonizes the action of Sox10 in specification of both these cell types.

### Sox10 and Sox5 cooperatively promote xanthophore formation

**Fig 3:**
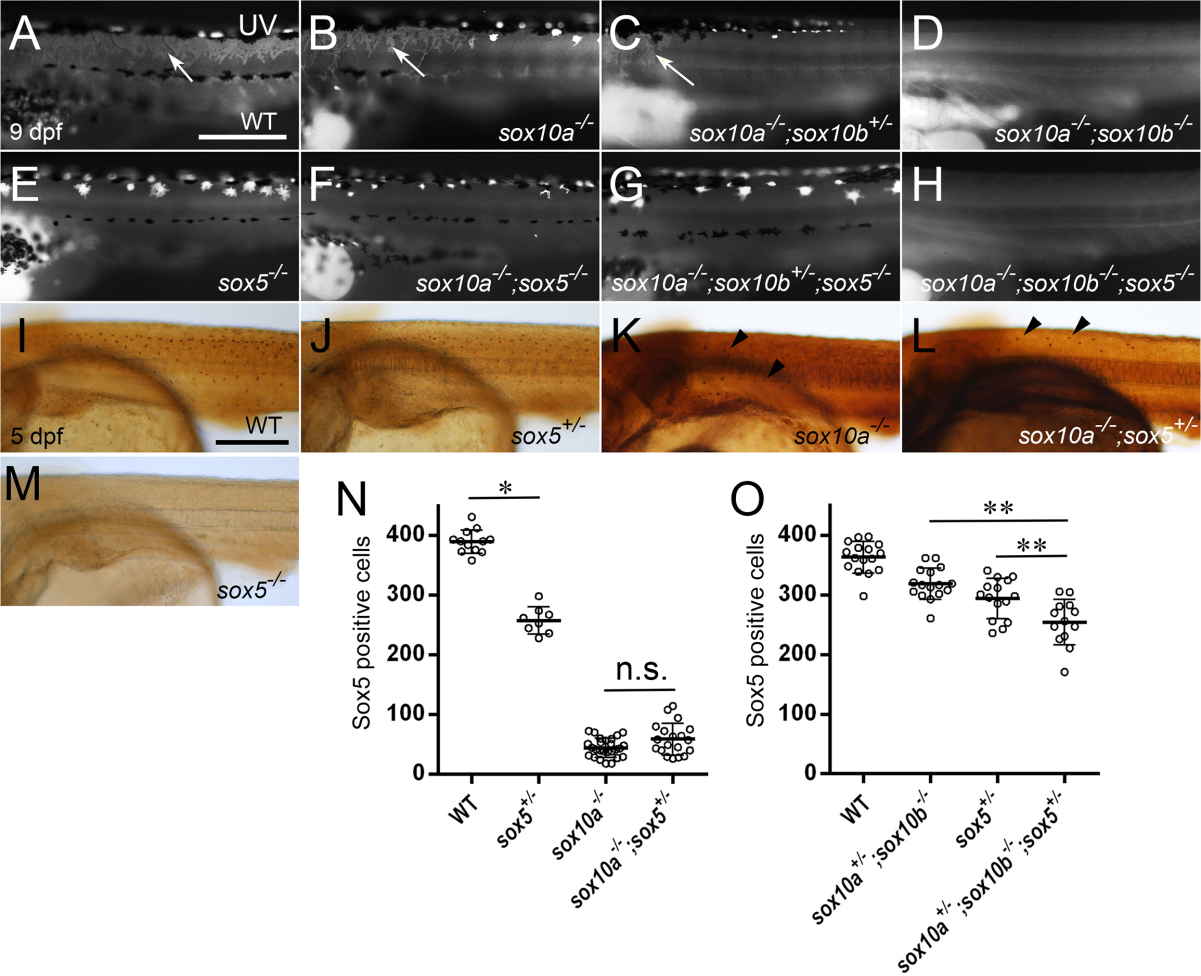
Cooperative function of Sox10 and Sox5 is required for xanthophore formation (A-H) 9 dpf hatchlings. Lateral views. UV images. (I-M) 5 dpf embryos. In WT (A), xanthophores (white arrows) are widely scattered under the skin, concentrated in the dorsal body. Xanthophores are reduced in *sox10a*^*-/-*^ mutants (B), and further reduced in *sox10a*^*-/-*^*;sox10b*^*+/-*^ mutants while a few residual cells remain in the anterior body (C). *sox10a*^*-/-*^*;sox10b*^*-/-*^ mutants completely lack xanthophores (D). Loss of *sox5* function results in complete absence of xanthophores, regardless of *sox10* mutant status (E-H). Xanthoblasts immunostained with anti-Sox5 antibody (I-L) were quantified in the body. (M) Specific signals cannot be detected in *sox5*^*-/-*^ embryos. *sox5*^*+/-*^ embryos (J) have significantly fewer Sox5-positive xanthoblasts than WT (I) (N, WT, n = 12; *sox5*^*+/-*^, n = 8, *p<0.05). Further reduction is observed in *sox10a*^*-/-*^ mutants (K) (N, n = 26). The number in *sox10a*^*-/-*^*;sox5*^*+/-*^ mutants (L) (N, n = 19) is comparable to *sox10a*^*-/-*^ embryos (*p* = 0.288). Comparison between the genotypes was performed by Kruskal-Wallis test with Steel-Dwass-Critchlow-Fligner (SDCF) post hoc test. The compound mutant of *sox10a*^*+/-*^*;sox10b*^*-/-*^ results in reduced numbers of Sox5-positive xanthoblasts compared to WT (O, WT, n = 16; *sox10a*^*+/-*^*;sox10b*^*-/-*^, n = 16). In *sox10a*^*+/-*^*;sox10b*^*-/-*^*;sox5*^*+/-*^ mutants (n = 13), the number of Sox5-positive cells is reduced compared to both *sox5*^*+/-*^ (n = 15) and *sox10a*^*+/-*^*;sox10b*^*-/-*^ mutants (O). Comparison between the genotypes was performed by Kruskal-Wallis test with SDCF post hoc test. **, *p*<0.05. (N, O) Bars show mean and error bar (s.d.). Scale bars: (A, I) 200 μm.

We then asked how xanthophore specification was affected in *sox10* mutants and how this was affected by loss of Sox5 activity. Xanthophore phenotypes were observed in hatchlings, using a combination of the distinctive auto-fluorescence, morphology and location of these cells (Fig 3A–3H). Compared with WT (Fig 3A), *sox10a*^*-/-*^ mutants had reduced numbers of xanthophores in the body (Fig 3B). In *sox10a*^*-/-*^*;sox10b*^*+/-*^ mutants, reduction was more severe (Fig 3C), and *sox10a*^*-/-*^*;sox10b*^*-/-*^ double mutants completely lacked xanthophores (Fig 3D), suggesting that xanthophore formation depends quantitatively on the number of functional *sox10* alleles. Loss of *sox5* caused complete absence of xanthophores regardless of the mutation status of *sox10a* and *sox10b* (Fig 3E–3H). Thus, both Sox5 and Sox10 are essential for xanthophore development.

In order to assess genetic interaction between Sox5 and Sox10 in the xanthophore lineage, we combined the *sox10* mutant alleles with the heterozygous *sox5* mutation. Xanthophore number was examined in 5 dpf embryos using immunohistochemistry with anti-Sox5 antibody, which marks the xanthophore lineage at this stage. Validation of the Sox5-specific nature of this antibody was shown by immunohistochemistry of *sox5*^*-/-*^ embryos, which showed the expected absence of specific signal (Fig 3M). We used this anti-Sox5 antibody in double immunofluorescent studies to assess the overlap between Sox5 and Pax7 expression (S5 Fig). We find that at 90 hpf, Pax7-expressing cells are of two classes, those showing Sox5 expression and those without. The former are mainly on the lateral migration pathway and are interpreted as xanthoblasts, whereas the latter can be interpreted as leucoblasts, based on our previous observations that *pax7a* mRNA is expressed in both the xanthophore and leucophore lineage, but that *sox5*^*-/-*^ mutants lack laterally migrating *pax7a-*expressing cells [18].

We then used the Sox5 antibody to assess the phenotypes of the *sox10* and *sox5* mutants. Consistent with our previous result that fewer fluorescing xanthophores could be seen in 5 dpf *sox5*^*+/-*^ mutants than in WT [18], Sox5-positive cells were decreased in the 5 dpf mutants compared to WT embryos (Fig 3I, 3J and 3N). In *sox10a*^*-/-*^ mutants as well as in *sox10a*^*-/-*^*;sox5*^*+/-*^ mutants, Sox5-positive xanthoblasts were severely reduced (Fig 3K and 3L), and there was no significant difference between these mutants (Fig 3N). We then assessed xanthoblasts in a weaker background; *sox10a*^*+/-*^*;sox10b*^*-/-*^ mutants showed significant reduction in xanthoblasts compared to WT siblings (Fig 3O). Reduction of a *sox5* WT allele led to further reduction of xanthophores in the *sox10a*^*+/-*^*;sox10b*^*-/-*^ mutants (Fig 3O). These data indicate that *sox5* and *sox10* additively promote xanthophore formation in medaka.

### Sox10 and Sox5 repress leucophore development

**Fig 4:**
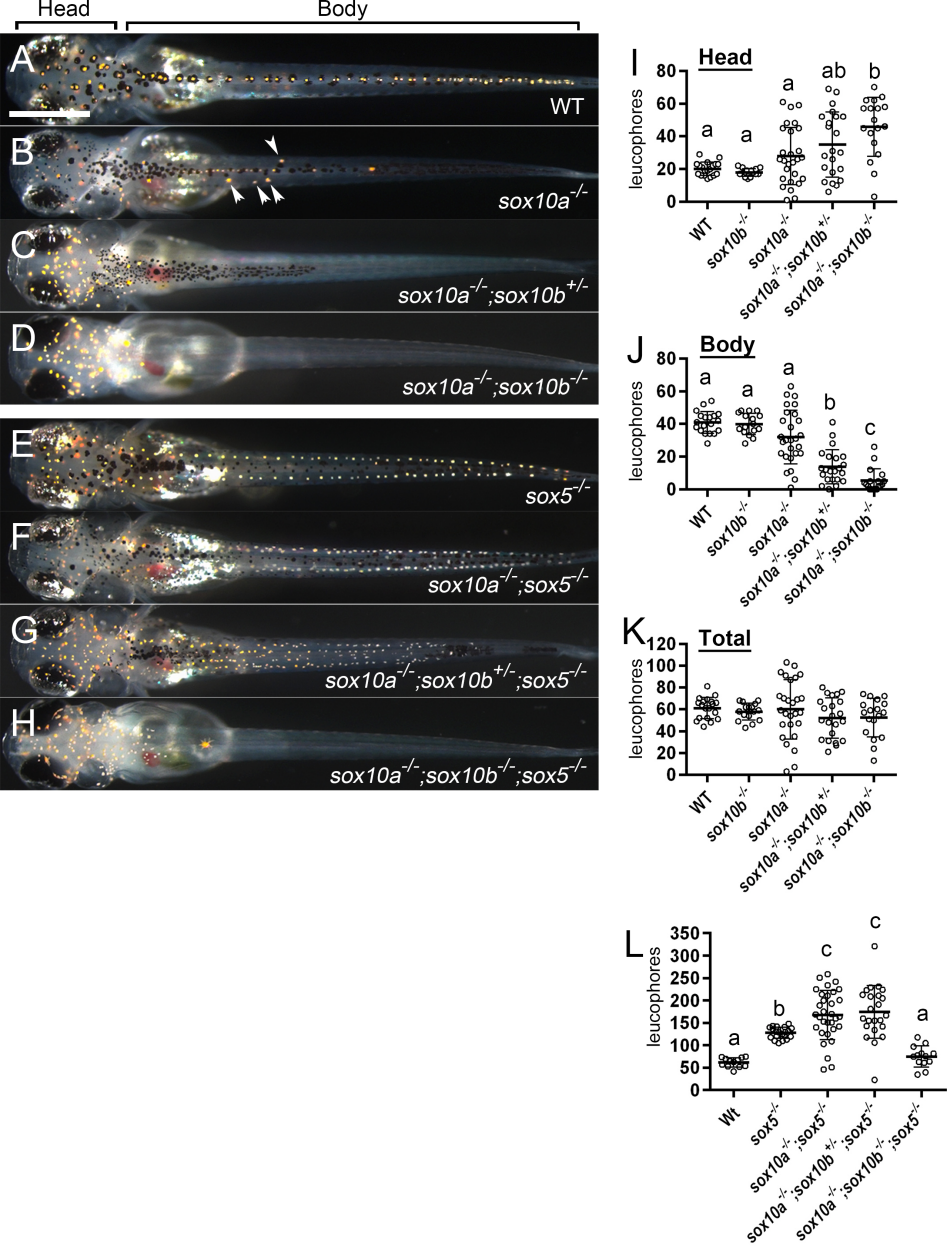
Leucophore formation does not require Sox10 function but is repressed by Sox10 and Sox5 (A-H) 9 dpf. Dorsal views. Leucophores (orange) are distributed along the dorsal surface throughout the anterioposterior axis of WT, as scattered individual cells in the head, and along the midline in the body (A). As the number of functional *sox10* alleles decreases, leucophore numbers on the head are progressively increased (A-D, I, *p*<0.05 by Kruskal-Wallis test), whereas in the body they are progressively decreased (J, *p*<0.05 by Kruskal-Wallis test). (I, J) Comparison between genotypes was performed by Kruskal-Wallis test with SDCF post hoc test. Statistical significance (*p*<0.05) was detected between the groups shown by letters (a, b or c) above each scatter plots. *sox10a*^*-/-*^ mutants have some ectopic leucophores on dorsal trunk (B, arrowheads). In *sox10a*^*-/-*^*;sox10b*^*+/-*^ mutants, trunk leucophores are restricted to the anterior dorsal region (C). In *sox10a*^*-/-*^*;sox10b*^*-/-*^ mutants, leucophores are largely restricted to the head (D). Intriguingly, the total number of leucophores in the whole body is not statistically different among these genotypes (K, *p* = 0.51 by Kruskal-Wallis test), although numbers do become much more variable after loss of *sox10a*. (I-K) WT, n = 19; *sox10b*^*-/-*^, n = 16; *sox10a*^*-/-*^, n = 27; *sox10a*^*-/-*^*;sox10b*^*+/-*^, n = 22; *sox10a*^*-/-*^*;sox10b*^*-/-*^, n = 18. In *sox5*^*-/-*^ mutants, leucophores are formed in excess (L) and scatter more laterally (E). In *sox10a*^*-/-*^*;sox5*^*-/-*^ mutants (F), there is a further increase in leucophores, which are scattered laterally like in *sox5*^*-/-*^ mutants (E). Abundant leucophores (greater than in *sox5*^*-/-*^ mutants; L) are observed in *sox10a*^*-/-*^*;sox10b*^*+/-*^*;sox5*^*-/-*^ mutants, but these are more scarce in the body from posterior trunk backwards (G). In *sox10a*^*-/-*^*;sox10b*^*-/-*^*;sox5*^*-/-*^ mutants, most leucophores are located on the head, but with some in the anterior trunk (H). (L) WT, n = 12; *sox5*^*-/-*^, n = 20; *sox10a*^*-/-*^*;sox5*^*-/-*^, n = 32; *sox10a*^*-/-*^*;sox10b*^*+/-*^*;sox5*^*-/-*^, n = 23; *sox10a*^*-/-*^*;sox10b*^*-/-*^*;sox5*^*-/-*^, n = 13. Comparison between the genotypes was performed by Kruskal-Wallis test with SDCF post hoc test. Statistical difference (*p*<0.05) was detected between the groups shown by letters (a, b or c) above each scatter plots. (I-L) Bars show mean and error bar (s.d.). Scale bar: (A) 200 μm.

We assessed leucophore development in *sox10* mutant hatchlings (Fig 4). Loss of Sox10 function had distinct effects in different locations. Although there was no significant difference in total leucophore numbers between WT and *sox10a*^*-/-*^ mutants (Fig 4K), leucophores decreased in the body and increased in the head upon loss of Sox10a (Fig 4A, 4B, 4I and 4J). Leucophores were significantly further reduced in *sox10a*^*-/-*^*;sox10b*^*+/-*^ mutants (Fig 4C and 4J) and more severely reduced in *sox10a*^*-/-*^*;sox10b*^*-/-*^ mutants in the body (Fig 4D and 4J). In contrast to the body, reduction of functional *sox10* alleles resulted in increasing numbers of leucophores on the dorsal surface of the head (Fig 4I). These surprising findings indicate that loss of Sox10 led to progressive decrease in leucophore formation in the body, but to progressive increase in the head. However, there was no difference in the total leucophore numbers between WT and *sox10* compound mutants (Fig 4K).

We then examined the effect of the loss of Sox10 on the *sox5*-mutant leucophore phenotype. As described previously [18], loss of Sox5 function alone caused significant increase in leucophores compared to WT (Fig 4E and 4L). Additional loss of *sox10a* and *sox10b* in the *sox5* mutants enhanced the leucophore phenotype: *sox10a*^*-/-*^*;sox5*^*-/-*^ and *sox10a*^*-/-*^*;sox10b*^*+/-*^*;sox5*^*-/-*^ mutants had more leucophores than *sox5*^*-/-*^ mutants (Fig 4F, 4G and 4L). The data suggest that Sox10 functions in parallel to Sox5 to repress leucophore formation. As *sox10a*^*-/-*^*;sox10b*^*-/-*^*;sox5*^*-/-*^ triple mutants had comparable numbers of leucophores to WT (Fig 4H and 4L), Sox10 is required for the Sox5-mediated suppression of leucophore development.

The differential effect of *sox10* on leucophores in the head and trunk is striking and paradoxical. In the trunk and tail, leucophores seem to behave like all other pigment cell-types, with numbers proportional to the number of functional *sox10* alleles, and there being none except the odd ‘escaper’ in the absence of any functional *sox10* alleles. In contrast, in the head, the pattern is the reciprocal, with leucophore number increasing progressively with loss of functional *sox10* alleles. One plausible explanation of this is that any maternal contribution of *sox10* might be most active in the head region, simply because the neural crest develops earlier in this region. Thus, in the head, but not in the trunk, we might expect rescue of the zygotic phenotype by maternally-supplied Sox10 activity. To test this idea, we used RT-PCR to examine medaka embryos at 2–4 cell stage (well-before mid-blastula transition), as well as at stage 25 (18–19 somite stage) when zygotic transcription is known to be active (S6 Fig). Consistent with our hypothesis, maternal contribution of *sox10a* and *sox10b* was readily detected, as indeed was *sox9a* and *sox9b*.

### In zebrafish Sox5 represses Sox10 function in all three pigment cell lineages

**Fig 5:**
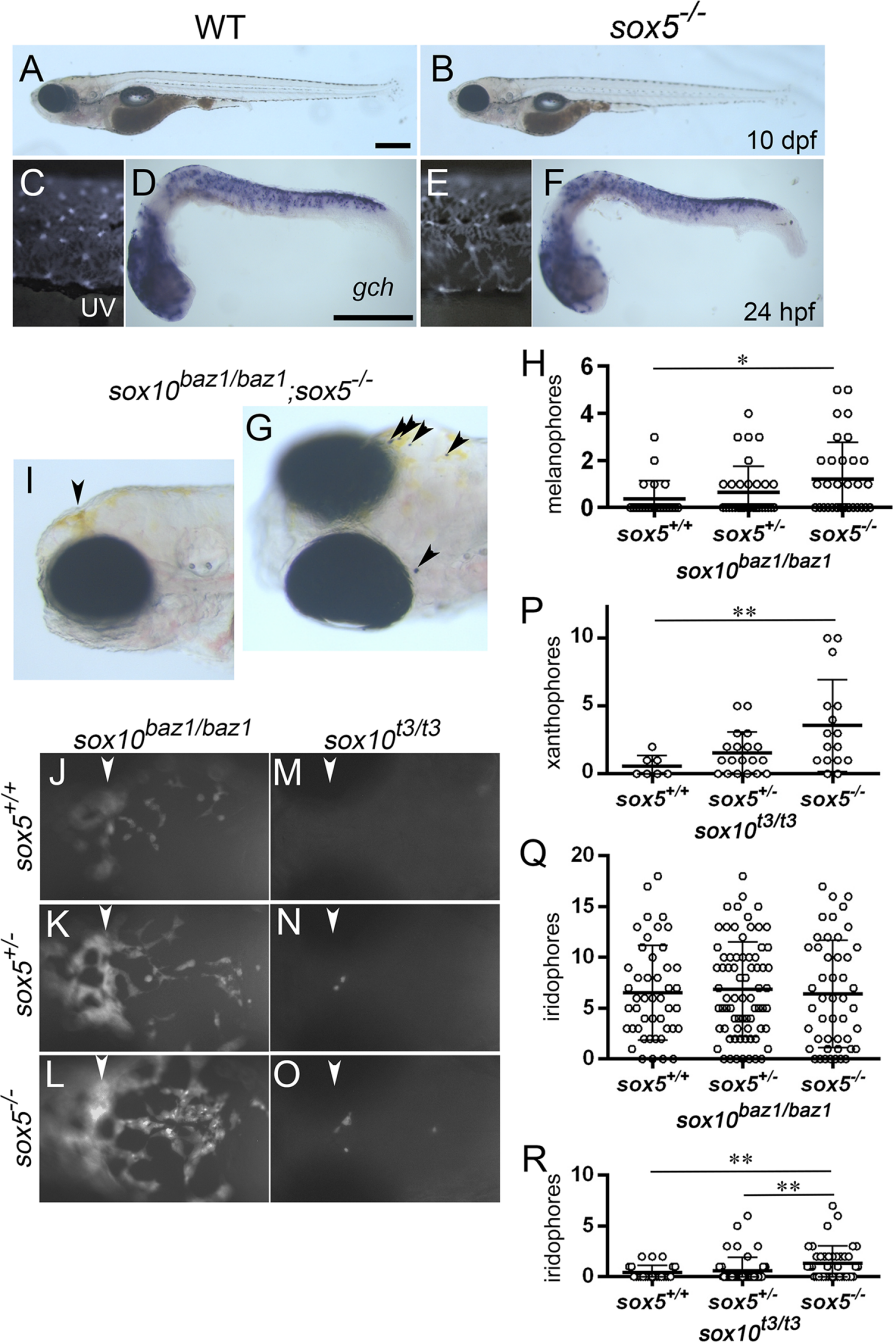
**Sox10-mediated pigment cell formation is modulated by Sox5 in zebrafish**. (A, B, C, E) Swim bladder inflation stage (10 dpf). Lateral views. (C, E) UV images. (D, F) 24 dpf. Lateral views. (G, I-O) 4 dpf. (G) Dorsolateral view. (I) Lateral view. (J-O) Fluorescing xanthophores. Dorsal views anterior to the left. The *sox5* mutant is indistinguishable from WT, exhibiting four stripes of melanocytes (A, B) and having fluorescing xanthophores (C, E) and *gch*-expressing xanthoblasts (D, F). Melanocytes are almost completely absent from *sox10*^*baz1/baz1*^ mutant (S8B Fig), but are partially recovered in *sox10*^*baz1/baz1*^ mutants that have also lost *sox5* WT allele(s) (G, H). The ratio of the embryos without melanocytes or with more than one melanocytes was compared among genotypes by Chi-squared test (*p<0.05). A few xanthophores develop on surface of the head in *sox10*^*baz1/baz1*^ mutant (S8E, S8I and S8J Fig), and reduction of *sox5* is likely to elevate xanthophore formation (K, L). Whereas *sox10*^*t3/t3*^ mutant almost completely lacks xanthophores (M), a few xanthophores are rescued as the *sox5* WT allele(s) are reduced (N, O). The counts are shown for xanthophores on the *t3* background (P). Comparison between the genotypes was performed by Kruskal-Wallis test with SDCF post hoc test. **p<0.05. A substantial number of iridophores are formed in *sox10*^*baz1/baz1*^ mutants (S8G and S8H Fig), and the counts are not significantly altered with reduction of the *sox5* WT allele(s) (Q, *p* = 0.775 by Kruskal-Wallis test). Iridophores are almost completely lost in *sox10*^*t3/t3*^ mutant (S8I Fig), but are partially recovered in *sox10*^*t3/t3*^*;sox5*^*+/-*^ and *sox10*^*t3/t3*^*;sox5*^*-/-*^ mutants (R **p<0.05 by Kruskal-Wallis test with SDCF post hoc test). (H) *sox10*^*baz1/baz1*^*;sox5*^*+/+*^, n = 22; *sox10*^*baz1/baz1*^*;sox5*^*+/-*^, n = 34; *sox10*^*baz1/baz1*^*;sox5*^*-/-*^, n = 33. (Q) *sox10*^*baz1/baz1*^*;sox5*^*+/+*^, n = 46; *sox10*^*baz1/baz1*^*;sox5*^*+/-*^, n = 73; *sox10*^*baz1/baz1*^*;sox5*^*-/-*^, n = 47. (P) *sox10*^*t3/t3*^*;sox5*^*+/+*^, n = 7; *sox10*^*t3/t3*^*;sox5*^*+/-*^, n = 19; *sox10*^*t3/t3*^*;sox5*^*-/-*^, n = 16. (R) *sox10*^*t3/t3*^*;sox5*^*+/+*^, n = 24; *sox10*^*t3/t3*^*;sox5*^*+/-*^, n = 48; *sox10*^*t3/t3*^*;sox5*^*-/-*^, n = 40. (H, P-R) Bars show mean and error bar (s.d.). Arrowheads point to weakly melanised cells (G) and at the corresponding position of the head (I-O). Scale bars: (A, D) 200 μm.

To understand the general role of Sox5 and Sox10 in pigment cell development in teleosts, we conducted similar genetic analyses with zebrafish mutants and compared their phenotypes with those of medaka. In zebrafish, *sox10* expression is prominent throughout the early neural crest from premigratory stages, and persists in cells on the medial and lateral migration pathways, before levels become gradually decreased in differentiating cells, with the exception of cartilage and peripheral glia which maintain strong expression [23, 30, 51, 52]. In contrast, *sox5* expression was detected in trunk neural crest at 18 hpf (S7A, S7C and S7E Fig), overlapping with *sox10* expression (S7B, S7D and S7F Fig), but seemed much weaker than in medaka. In an independent study using single cell transcriptional profiling, we have confirmed the expression of *sox5* in flow cytometry (FACS)-purified neural crest cells, with highest expression corresponding to likely premigratory neural crest cells (Subkhankulova et al., in preparation). It is noteworthy that *sox5* expression was transient and faint in zebrafish whereas in medaka it is retained in migrating xanthophore precursors [18]. We established a novel *sox5* mutant (*sox5*^*E4del7*^) in zebrafish by using the CRISPR/Cas9 system [53] (S2 Fig). Homozygous *sox5*^*E4del7*^ mutants (referred to as *sox5*^*-/-*^ below) showed normal pigment cell pattern during larval stages (Fig 5A and 5B, shown for swim bladder inflation stage [54]). Unlike medaka *sox5* homozygotes, zebrafish *sox5*^*-/-*^ mutants did not show apparent changes in the formation of xanthophores (Fig 5C and 5E) nor of xanthophore precursors expressing *GTP cyclohydrolase 1* (*gch*) [55] (Fig 5D and 5F).

In order to test genetic interaction between *sox5* and *sox10* in zebrafish, we generated *sox5;sox10* double mutants. We employed two zebrafish mutant alleles: *colourless/sox10*^*t3*^ as a likely null allele exhibiting an absence of normal melanocytes with severe defects in xanthophores and iridophores [29, 30] and *sox10*^*baz1*^ allele, which has a single nucleotide change causing an amino acid substitution (V117M) in the HMG box domain (S2 Fig) and is a hypomorphic mutant allele [23, 51] (S8 Fig). We hypothesized that given that the Sox10^*baz1*^ mutant protein retains weak Sox10 activity, *sox10*^*baz1*^ mutants give a condition more similar to medaka *sox10a*^*-/-*^ or *sox10a*^*-/-*^*;sox10b*^*+/-*^ mutants. Most *sox10*^*baz1/baz1*^ mutants had no melanocytes except for some visible simply as tiny residual specks of melanin, while a small minority had a few partially-differentiated melanocytes on the head (Fig 5G). Loss of *sox5* in *sox10*^*baz1/baz1*^ mutants resulted in an increased number of partially-differentiated melanocytes (Fig 5G and 5H). *sox10*^*baz1/baz1*^ mutants have some visible xanthophores on the head and very few xanthophores in the trunk (Figs 5I, 5J, S8E and S8E’). Reduction of *sox5* WT alleles in *sox10*^*baz1/baz1*^ mutants appears to result in increasing numbers of xanthophores, but we were not able to quantify xanthophore formation in *sox10*^*baz1/baz1*^ and *sox5*^*-/-*^*;sox10*^*baz1/baz1*^ mutants due to their dense localization on the dorsal head (Fig 5J–5L). In *sox10*^*t3/t3*^ mutants, which have no or only a few fluorescing xanthophores, reduction of *sox5* WT alleles significantly increased xanthophore numbers (Fig 5M–5P). These results indicate that Sox5 acts to repress Sox10-dependent melanocyte and xanthophore formation. Whereas we were unable to demonstrate a significant alteration in residual iridophores in *sox10*^*baz1/baz1*^ mutants that had also lost one or both *sox5* alleles, *sox5* allele loss in *sox10*^*t3/t3*^ mutants rescued iridophores to a small but significant extent (Fig 5Q and 5R). Taken together, these data show that Sox5 antagonizes Sox10 function in development of all three pigment cell types in zebrafish.

### Role of Sox5 in adult xanthophore development in medaka remains opposite to that in zebrafish

**Fig 6:**
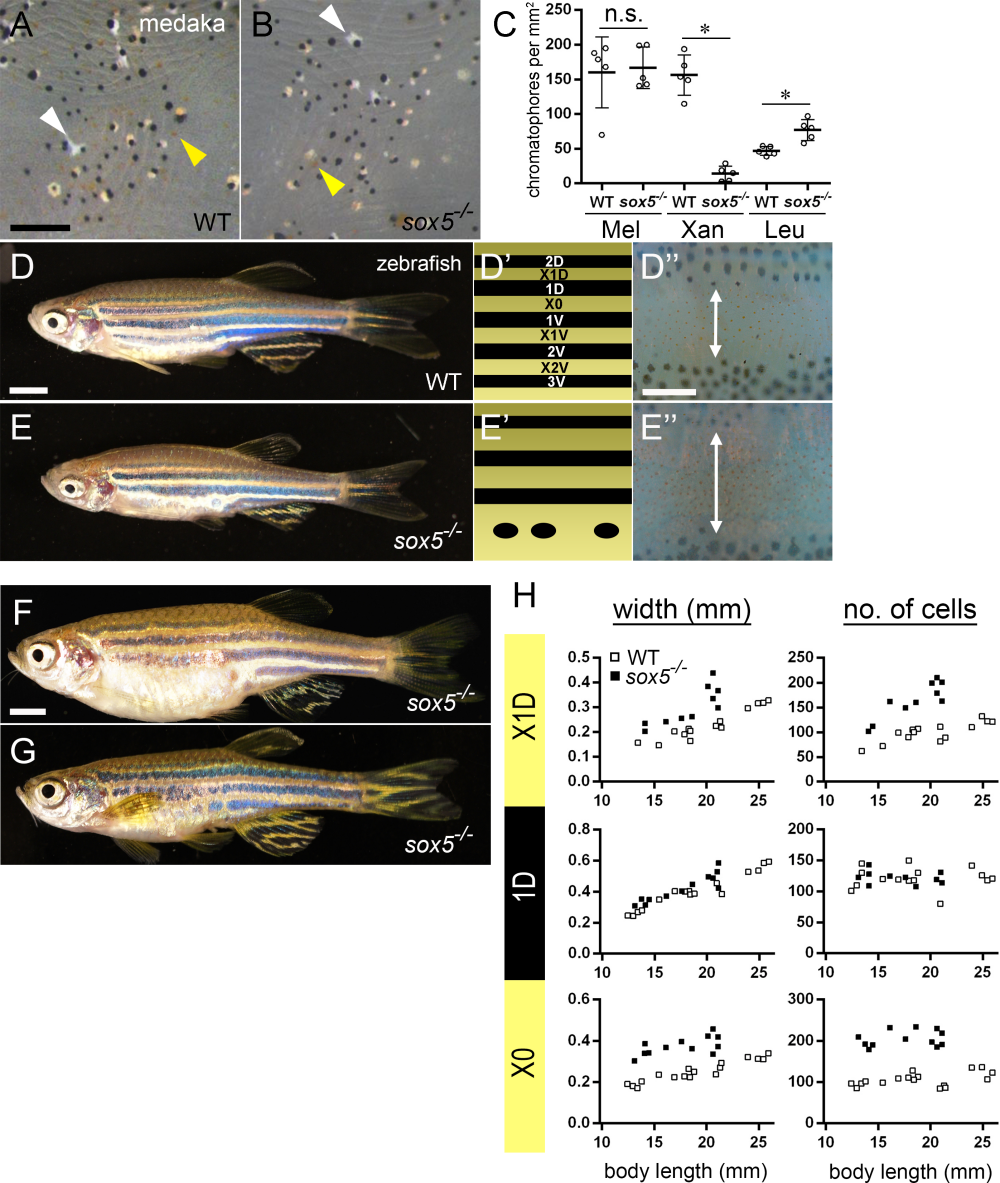
**Role of Sox5 in adult xanthophore development in medaka remains opposite to that in zebrafish**. (A, B) Medaka. 6 mpf. (D, E) Zebrafish. 2 mpf. (D’, E’) Schematics of stripe pattern. (D”, E”) Enlarged images of X0 interstripe. (F, G) Zebrafish. 1.5 years old. In WT medaka (A), melanocytes, xanthophores (yellow arrowhead) and leucophores (white arrowhead) are scattered on the body surface. *sox5*^*-/-*^ mutant medaka (B) have fewer xanthophores and more leucophores than WT (C, Xan and Leu, *p<0.05 by Mann-Whitney test; WT, n = 5; *sox5*^*-/-*^, n = 5; Bars show mean and error bar (s.d.)). Pigment cells were counted from a 1 mm^2^ area on the dorsal body surface. The melanocyte numbers were not significantly different between WT and *sox5*^*-/-*^ (C, Mel, *p* = 0.1 by Mann-Whitney test). The adult pigment pattern of WT zebrafish is composed of 5 melanocyte stripes (2D, 1D, 1V, 2V, 3V) and xanthophore interstripes (X1D, X0, X1V, X2V) (D, D’). Zebrafish *sox5*^*-/-*^ mutants lack two ventral interstripes (2V and 3V) (E, E’), and thus have fewer stripes than WT. This is the case regardless of sex (F, female; G, male) after the mutant fish get larger and older than 1.5 years. The *sox5*^*-/-*^ zebrafish mutants have wider X1D and X0 (two-way arrow in D” and E”) interstripes and larger numbers of xanthophores in these interstripes than WT. (H) Scatter plot of stripe width or pigment cell numbers in each stripe, comparing *sox5*^*-/-*^ and their WT siblings. X axis shows the standard body length of zebrafish examined. Analysis of covariance was performed to examine the differences in width or cell numbers between WT and *sox5*^*-/-*^ mutants, by using standard length as a covariate. The *p* values are as follows; width X1D (*p*<0.05), 1D (*p*<0.05), X0 (*p*<0.05), cell number X1D (*p*<0.05), 1D (*p* = 0.985), X0 (*p*<0.05). The width and number of xanthophore in the xanthophore stripes (X0, X1D) in *sox5*^*-/-*^ mutants (black boxes) show significant increase compared with WT siblings (white boxes). The number of melanocyte in the 1D stripe is comparable between WT (white boxes) and *sox5*^*-/-*^ mutant (black boxes), but the width is slightly but significantly different (*p*<0.05). Scale bar: (A) 200 μm, (D) 3 mm, (D”) 200 μm, (F) 3 mm.

Medaka *sox5* homozygotes survive to adulthood and look normal except that they show an increase of leucophore number and decrease of xanthophore number (Fig 6A–6C). Thus, the function of Sox5 in adult xanthophore and leucophore development is reminiscent of that at embryonic stages. Zebrafish *sox5*^*-/-*^ mutants looked normal at larval stages, but had fewer horizontal melanocyte stripes at 60 dpf. Whereas WT zebrafish had five stripes, *sox5*^*-/-*^ mutant had three to four stripes (Fig 6D–6G). We examined post-metamorphic phenotypes of xanthophores and melanophores in *sox5*^*-/-*^ mutant at juvenile to adult stages [54]. The width of melanocyte stripe (1D) is marginally, but significantly larger in *sox5*^*-/-*^ mutants compared to WT siblings, and the xanthophore interstripes (X0 and X1D) were significantly wider in *sox5*^*-/-*^ mutants than in WT (Fig 6D”, 6E” and 6H). We found that each interstripe in *sox5*^*-/-*^ mutants contains an increased number of xanthophores but the melanocytes number in melanocyte stripe was not altered (Fig 6H). These results suggest that adult zebrafish xanthophore formation is upregulated by loss of Sox5 activity. Thus, Sox5 acts to repress adult xanthophore formation, similar to its role in xanthophore development at embryonic/larval stages, as observed in a *sox10*-defective sensitive background. Our data also indicate the contrasting role of Sox5 in xanthophore development between medaka (promoting) and zebrafish (repressing).

### Sox5 misexpression in the shared xanthophore/leucophore progenitors promoted the xanthophore and repressed the leucophore lineage

**Fig 7:**
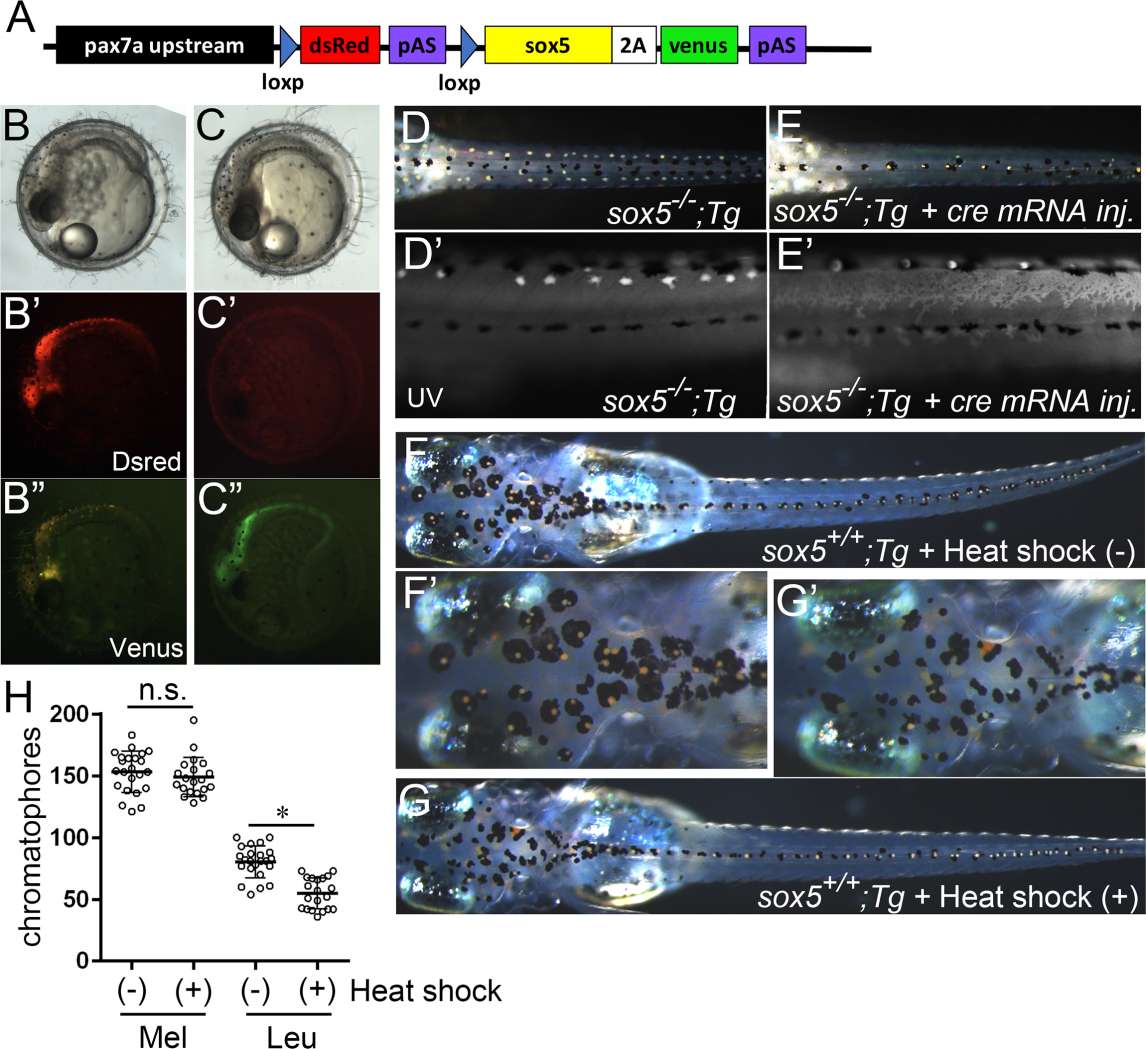
**Misexpression of Sox5 can reverse the xanthophore vs leucophore fate choice in medaka**. (A) Transgenic construct. (B-C”) 3 dpf. (B, C) Bright field. (B’, C’) Dsred. (B”, C”) Venus. (D-G) 7 dpf. Dorsal views. (D’, E’) lateral views. UV images. (F’, G’) Magnified images of the head in (F, G). (B, D) *sox5*^*-/-*^;*Tg(pax7a*:*loxp-dsred-loxp-sox5*^*WT*^*)*. (C, E) *sox5*^*-/-*^*;Tg(pax7a*:*loxp-dsred-loxp-sox5*^*WT*^*)* injected with *cre* mRNA. (F) WT(*sox5*^*+/+*^);*Tg(pax7a*:*loxp-dsred-loxp-sox5*^*WT*^*);Tg(hsp70*:*cre)* without heat shock. (G) WT;*Tg(pax7a*:*loxp-dsred-loxp-sox5*^*WT*^*);Tg(hsp70*:*cre)* with heat shock. A transgenic fish in which the *pax7a* promoter drives *sox5-2A-venus* expression once the upstream *dsred* region is excised at the flanking loxp sites by Cre recombinase *TgBAC(pax7a*:*loxp-dsred-loxp-sox5*^*WT*^) was used for Sox5 misexpression in a shared xanthophore/leucophore progenitor. In the absence of *cre* mRNA, *sox5*^*-/-*^*;Tg* embryos (*sox5*^*-/-*^;*TgBAC(pax7a*:*loxp-dsred-loxp-sox5*^*WT*^)) were strongly expressing Dsred (B-B”, n = 8). When *cre* mRNA was injected in the 1-cell *sox5*^*-/-*^*;Tg* embryos, they became positive for Venus (C-C”, n = 7). Venus-expressing animals showed rescued xanthophore and leucophore phenotypes in *sox5*^*-/-*^ mutants (D, E). When *sox5*^*+/+*^ WT embryos obtained from crossing the transgenic fish Nagoya (*sox5*^*+/+*^);*TgBAC(pax7a*:*loxp-dsred-loxp-sox5*^*WT*^*)* with *Tg(hsp70*:*cre)* were heat shocked, Dsred became barely detectable. Leucophores (Leu) were significantly reduced in the hatchlings treated with heat shock (+) compared with those without heat shock (-) whereas melanocyte (Mel) formation was not altered by heat shock (F-H). (H) Bars show mean and error bar (s.d.). Comparison between with and without heat shock was performed by paired t-test (*p<0.0001).

The difference in the genetic interactions between *sox10* and *sox5* in xanthophore development medaka and zebrafish were surprising, but they correlate with another important difference in the pigment cells of these fish species, specifically the presence of leucophores in medaka, but not in zebrafish. In previous work we and others have shown that leucophores are best considered as a modified form of xanthophore, and that the key distinction correlates with maintenance of *sox5* expression in cells that will become xanthophores [18]. Importantly, our previous data suggested that maintenance of *sox5* function in shared xanthophore/leucophore progenitors was incompatible with leucophore development, although we had not directly tested this hypothesis. In the context of the data presented here showing that xanthophore formation in zebrafish does not require Sox5 activity, we suggest that medaka Sox5 may have acquired an evolutionarily novel function in driving xanthophore development, associated with the evolution of the related leucophore cell-type. To test the role of Sox5 function in promoting the xanthophore and repressing the leucophore lineages, we generated medaka transgenic lines *TgBAC(pax7a*:*loxp-dsred-loxp-sox5*^*WT or ml-3*^*)* that misexpress either WT or *sox5*^*ml-3*^ mutant form of Sox5 protein in the shared xanthophore/leucophore progenitors (controlled by use of *pax7a* promoter) when in the presence of Cre recombinase (excises *dsRed* cDNA at the loxp sites upstream of *sox5* cDNA; Fig 7A–7C). When *Cre* mRNA was injected into *sox5*^*-/-*^;*TgBAC(pax7a*:*loxp-dsred-loxp-sox5*^*WT*^*)* embryos, xanthophore formation was restored in the hatchlings (Fig 7D’ and 7E’) while excessive formation of leucophores was suppressed (Fig 7D and 7E). We also expressed *Cre* mRNA using an *hsp70* promoter in *Tg(hsp70*:*Cre)* line. When *TgBAC(pax7a*:*loxp-dsred-loxp-sox5*^*WT*^*)*;*Tg(hsp70*:*cre)* embryos were treated with heat shock at the end of gastrulation, the hatchlings exhibited decreased leucophore numbers compared to those without heat shock (Fig 7F–7H). The misexpression of Sox5^ml-3^ mutant protein failed to alter the formation of leucophores at the hatching stage (S9 Fig). These results support the hypothesis that Sox5 in medaka has evolved a novel, major role in driving xanthophore fate and repressing leucophore fate within the *pax7a-*expressing shared progenitors in medaka that is coupled to the evolution of the novel leucophore cell-type.

## Discussion

Fate specification of neural crest cells is a key aspect of their development, and a model for stem cell fate choice. Fate specification of pigment cells forms a nice ‘model within a model’, but has an interesting added complexity in fish since the neural crest cells give rise to a variety of pigment cell types, and these vary between species. Our present study sought to dissect the mechanisms of pigment cell fate specification and their evolution, focusing on the genetic interactions of *sox5* and *sox10* in medaka and zebrafish.

Taking advantage of duplicated s*ox10* genes in medaka, we were able to produce a variety of *sox10* dosages in medaka and thereby a wide range of phenotypes in pigment cells. Although the role is shared by the two *sox10* genes, Sox10 function is required for specification of iridophores, xanthophores and melanocytes in medaka, just as in zebrafish (and in mammals) [25, 30, 32, 33, 56]. Thus, the mutant phenotypes are enhanced by simultaneous loss of *sox10a* and *sox10b*, and given their overlapping expression this clearly suggests functional redundancy of *sox10a* and *sox10b* in pigment cell specification. However, since medaka *sox10a* single mutants exhibited significant decrease in numbers of melanoblasts, iridoblasts and xanthophores, whereas *sox10b* single mutants appeared normal, medaka Sox10a seems to play a dominant role for specification of these pigment cells whereas Sox10b seems dispensable.

In contrast to the other three pigment cell types, medaka *sox10a*^*-/-*^*;sox10b*^*-/-*^ double mutants retained a considerable number of leucophores, especially on the head. Given that the mean *total* number of leucophores is apparently unaffected in *sox10* mutant medaka, it is conceivable that leucophore development from the neural crest is completely independent of SoxE function, although to explain the shift in the distribution towards the head as *sox10* allele number decreases, this hypothesis would also necessitate that endogenous leucophores in WT embryos derive from the head NC and then migrate posteriorly to populate the trunk and tail. However, given the essential role for Sox10 in the specification of all non-ectodermal fates including pigment cells in zebrafish (reviewed in [23]), we prefer the more conservative hypothesis that the development of leucophores may simply be achieved using lower levels of SoxE activity, perhaps especially Sox9b, similar to that shown in zebrafish where additional loss of *sox9b* results in loss of the residual melanocytes and sensory neurons of the DRGs in *colourless/sox10* mutants [23, 52]. To explain the shift in leucophore distribution towards the head as the numbers of functional *sox10* alleles are decreased, we propose that either a maternal contribution of SoxE proteins has a preferential effect on the earlier differentiating head NC or that Sox9b expression in the early NC is sufficient for specification of leucophores, but much less so for other pigment cell types; as the numbers of mutated *sox10* alleles increases, so more and more of the head NC remain unspecified and so remain vulnerable to the residual Sox9b activity. Consistent with both of these latter hypotheses, we were able to show the presence of detectable levels of all four *sox10* and *sox9* gene transcripts in the early cleavage stage zygote, demonstrating maternally supplied *soxE* activity. We note the highly variable nature of leucophore numbers in the *sox10* mutants, with some fish having essentially no leucophores, which suggests that leucophore specification is likely to be Sox10-dependent, but can be rescued in an unreliable manner in the head NC. Future study of medaka *sox10a;sox10b;sox9b* triple homozygous mutants would allow further testing of these ideas.

Our data suggests that, in medaka and zebrafish, Sox5 function has similar effects in development of both embryonic and adult metamorphic pigment cells, but that the relative importance of Sox5 in each stage varies between species. In medaka *sox5* mutant adult fish have reduced xanthophores and increased leucophores, which reflects the embryonic/larval phenotypes. However, in zebrafish, loss of Sox5 did not apparently affect the formation of any chromatophore types at embryonic/larval stages, indicating that Sox5 is dispensable for the development of embryonic pigment cells in zebrafish, similar to what has been shown in mice [41]. On the other hand, *sox5*-deficient adult zebrafish exhibited a clear phenotype, with increased numbers of xanthophores, but little effect on melanocytes, resulting in expanded interstripes with increased xanthophores and normal melanophore stripes.

While Sox5 function is maintained after metamorphosis, our data clearly demonstrate contrasting roles for Sox5 activity on xanthophore fate specification in medaka and zebrafish, being absolutely required for xanthophore specification in medaka, but weakly antagonizing xanthophore formation in zebrafish. Although Sox5 has no known transcriptional regulatory domains, it has been shown to collaborate with other transcription factors to regulate expression of downstream target genes [38–41]. We speculate that in medaka and zebrafish, Sox5 is likely to interact with different partner transcription factors controlling xanthophore specification, or to have differential affinity with similar partners in each species, which subsequently results in constructing different gene regulatory networks and thus establishing species-specific transcriptional regulation of target genes.

Sox5 counteracts Sox10 activity in the specification of melanocytes, iridophores and xanthophores in zebrafish, and of melanocytes and iridophores in medaka. Whereas loss of Sox5 alone had little impact on the formation of these chromatophore types, it partially rescued the severe reduction of these pigment cells due to Sox10 deficiency, revealing a subtle antagonistic function of Sox5 against Sox10. This mode of Sox5 and Sox10 interaction has been reported in mouse melanocytes and in human melanoma cells [41, 57], suggesting that an antagonistic interaction between Sox5 and Sox10 is widely conserved among vertebrates. In melanocytes, Sox5 antagonism likely functions through direct competition with Sox10 binding at a key regulatory binding site [41], but mechanisms seem to be varied and case-specific [37], so detailed investigation will be required to ascertain those functioning in each pigment cell case identified here.

**Fig 8:**
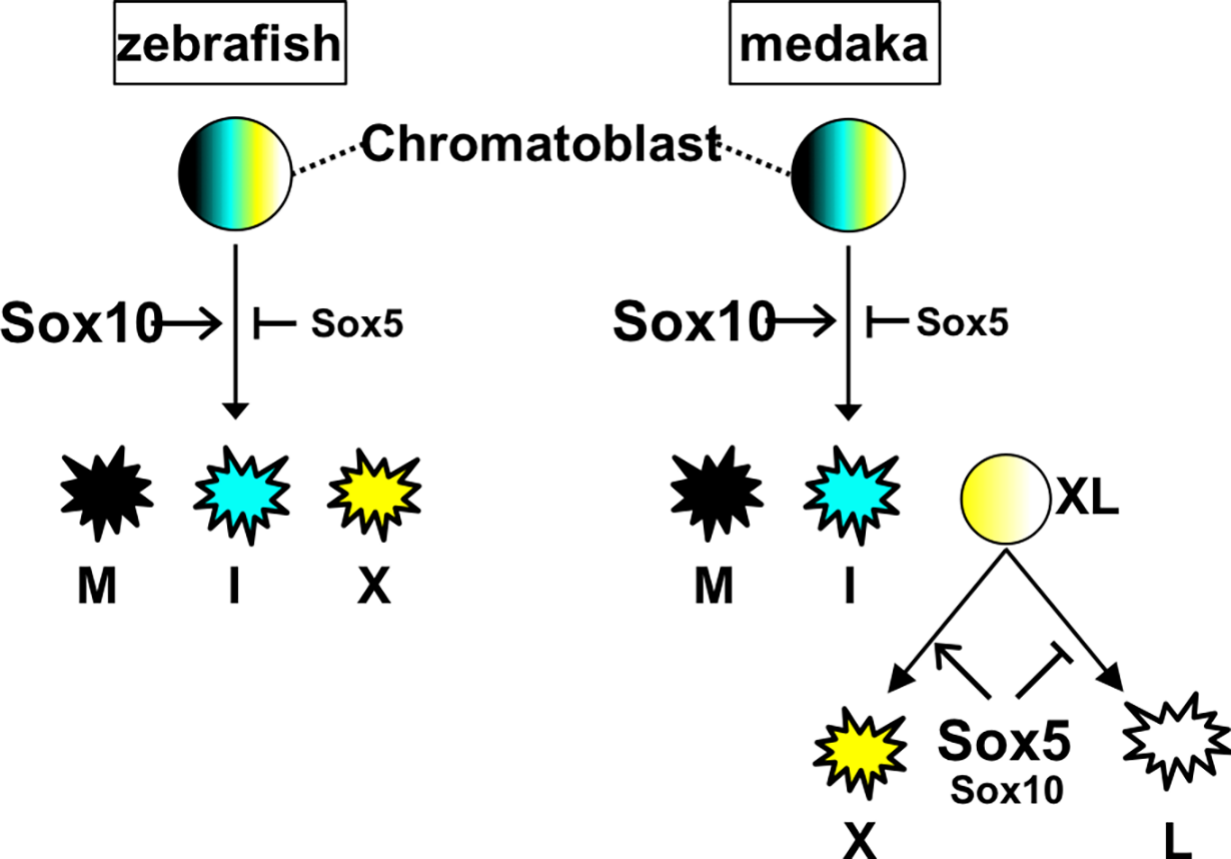
Based on a new working model for pigment cell development in medaka and zebrafish based on our previous work in zebrafish [46], we propose that a chromatoblast, a multipotent common progenitor of chromatophores, gives rise to three pigment cell types, melanocytes (M), iridophores (I) and xanthophores (X) in zebrafish, development of which is all dependent on SoxE with antagonistic modulation by Sox5. Likewise in medaka, Sox5 counteracts SoxE in the melanocyte and iridophore lineages and, by extension, presumably in formation of xanthophore/leucophore progenitor (XL). In an additional step, an evolutionary novelty of medaka, Sox5 likely functions in collaboration with Sox10 to promote xanthophore specification, but to repress leucophore (L) formation, from a novel shared progenitor.

In contrast, Sox5 and Sox10 function collaboratively in specification of xanthophores in medaka. Since *sox5* is a member of a group of related (*SoxD*) genes, it is conceivable that a SoxD function in zebrafish xanthophores is performed by another member of this group; however current expression data provide no evidence for this, so that this question is ripe for future investigation (ZFIN, sox6; https://zfin.org/ZDB-GENE-081120-6, sox13; https://zfin.org/ZDB-GENE-100519-1). Furthermore, we suggest that there may be no reason to expect conservation of this function in xanthophores. This contrast in the role of Sox5 in xanthophore specification between medaka and zebrafish is striking in that it correlates with the fact that in medaka, but not in zebrafish, xanthophores appear to derive from a shared (bipotential) progenitor and Sox5 is expressed in that progenitor, but also maintained at high levels in the xanthophore lineage (Fig 3) [17, 18]. To explain the difference in the interactions between Sox5 and Sox10, we hypothesize that Sox5 functions independently in two aspects of xanthophore development (Fig 8). In the first of these xanthophore progenitors are formed from multipotent chromatoblasts under the control of antagonistic interactions between Sox5 and Sox10. In a second step, unique to medaka, the xanthophore progenitor has acquired xanthophore/leucophore bipotency, and Sox5 plays a dominant role in xanthophore fate choice, being absolutely required for xanthophore specification. As attested by the details of the *sox5* single mutant phenotypes, the role for Sox5 in the first process is relatively weak, whereas in the second step Sox5 is the dominant player. Consistent with this idea, we show here that forced *sox5* expression in the shared xanthophore/leucophore progenitor is sufficient to drive xanthophore fate and to repress leucophore fate. Although this effect is substantial, in our hands we did not achieve a complete switch of leucophores to xanthophores; we attribute this to the experimental conditions used, which mean that the duration of expression of Cre is limited, and the timing of heat shock-mediated Cre induction, when some neural crest cells may already have become specified to a leucophore fate and so might be expected to be in a state that was more resistant to the effects of sox5 re-expression.

Melanocytes, xanthophores and iridophores are widely-distributed amongst teleost lineages [4]. In contrast, to date leucophores have been identified in just a few species, including guppy (*Lebistes reticulatus*), Japanese flounder (*Paralichthys olivaceus*), darkbanded rockfish (*Sebastes inermis*), and killifish (*Fundulus heteroclitus*) [10–13, 23, 58, 59], and hence those in medaka are likely an evolutionary novelty compared to the shared common ancestor with zebrafish. Although leucophores have been considered to be closely related to iridophores since the appearance of both cell types is dominated by reflection of light due to intracellular purine-crystal accumulation, our previous work showed that leucophores share an ontogenetic origin with xanthophores i.e. they share a (bipotent) progenitor [17, 18]. Thus, we speculate that medaka evolved the leucophore by expanding the potency of a xanthophore progenitor and using loss of Sox5 expression to drive accumulation of intracellular purine crystals in a xanthophore-like cell. Future work will explore the changes in transcriptional targets and partner proteins of Sox5, and especially the development of cooperative partnership between Sox5 and Sox10, in order to provide insight into the acquisition of new cell types during neural crest evolution.

## Materials and methods

### Ethics husbandry and strains

Medaka and zebrafish care was approved by Nagoya University ethics committee and in accordance with local and Japanese ethical guidelines or by AWERB committee of University of Bath and in accordance with the Animals Scientific Procedures Act 1986 of the UK. Medaka wild type (WT) strain Nagoya and the mutant strain *sox5*^*ml-3*^ [18] were used. Medaka *sox9b*^*K136X*^ TILLING mutant was provided by NBRP medaka [60]. Zebrafish AB and the mutant strains *sox10*^*baz1*^ and *sox10*^*t3*^ [23, 30] were used. In some experiments, embryos were treated with 0.003% phenylthiourea to inhibit melanin synthesis.

### Cloning and sequence analysis

Putative medaka *sox10b* and *ltk* genes were identified in Ensemble (http://asia.ensembl.org/Oryzias_latipes/Info/Index): *sox10b*; ENSORLG00000014587, *ltk*; ENSORLG00000015434. An entire open reading frame (ORF) of *sox10b* and a partial fragment of *ltk* were amplified from cDNA of medaka Orange red strain using following primers: 5’-ATGTCCAGGGAGGAGCAGAGCCT-3’ (*sox10b-*forward), 5’-TCATGGCCGCGACAGAGTTGTGTA-3’ (*sox10b*-reverse), 5’-CTGCTCTACACTTCCTGTGTGCCTG-3’ (*ltk-*forward) and 5’-GGCAGATCTTCTGGGTGAGGAGAT-3’ (*ltk-*reverse). The amplified fragments were subsequently cloned into pTAC-2 vector (BioDynamics Laboratory Inc). A putative *sox10a* gene was identified by BLASTN against zebrafish Sox10 protein (AF402677) in Ensembl (http://asia.ensembl.org/Oryzias_latipes/Info/Index). First, the identified region was cloned and sequenced. To determine full cDNA transcript of *sox10a*, 3’- and 5’- RACE were performed using gene specific primers. The primer sequences are available on request. The entire ORF of *sox10a* was cloned using the primers; 5’-ATGTCCGGTGAGGAGCACAGCCT-3’ (forward) and 5’-TCAAGGTCTTGTGAGGGTGGTGTAAA-3’ (reverse). The full-length sequence of medaka *sox10a* cDNA has been deposited in GenBank (accession number MF276972).

### TILLING screening of medaka mutants

TILLING screening was performed as described [49]. The exon 1 of medaka *sox10b* gene, which includes a part of HMG box domain, was amplified for the screening using primers: 5’-GCAGAGCTGAGAATTCACAAA-3’ (forward) and 5’-ACCTGTCGGGTCTGTCA-3’ (reverse).

### TALEN-mediated mutagenesis

The designing and assembling of TALENs were performed as described [47] [61]. The exon 1 and exon 2 sequences of medaka *sox10a* and exon 1 of medaka *sox10b* was submitted to the TALE-NT 2.0 (https://tale-nt.cac.cornell.edu/). We selected the following sequences as candidates for *sox10a* exon 1-specific target: 5’-TTCTGAGTCGGAGCTGAGC-3’ (left, sense) and 5’-TGAGAGTGAGTGACCGTCC-3’ (right, antisense), *sox10a* exon 2-specific target: 5’-TGCTGAACGAGAACGACAA-3’ (left, sense) and 5’-TCCTCAGCCTCTCCGCCTC-3’ (right, antisense) and *sox10b* exon 1*-*specific target: 5’-TCTCAGAGGTCGAGCTC-3’ (left, sense) and 5’-TGGGAGCAGCTGTCATCC-3’ (right, antisense). The TALEN expression vectors were linearized with NotI (NEB) and then used as templates for capped mRNA synthesis using the mMessage mMachine SP6 kit (Life Technologies). Equal amounts of mRNAs were mixed and microinjected into a blastomere of 1-cell stage embryos.

### CRISPR/Cas9-mediated mutagenesis

To generate small guide RNA (sgRNA) targeting the exon 4 of zebrafish *sox5* gene, we constructed a pT7-gRNA vector (Addgene plasmid # 46759) which harbors annealed oligonucleotides with the following sequences; 5’-TAGGCATGGGTTCTGGCAACTT-3’ (sense), 5’-AAACAAGTTGCCAGAACCCATG-3’ (antisense) [62]. The vector was linearized with BamHI (NEB) and used as a template for sgRNA synthesis using T7 RNA polymerase (Promega). Cas9 mRNA was synthesized with mMessage mMachine SP6 kit (Life Technologies) on NotI-digested pCS2-hSpCas9 (Addgene plasmid #51815) [63]. One nl of the RNAs solution (300 pg/nl Cas9 mRNA and 200 pg/nl sgRNA) was injected in the blastomere of 1-cell stage embryos.

### Transgenic medaka strains

We constructed transgenic vectors using a BAC clone, which contains the entire exons of *pax7a* gene (ola-008A15, [17]). To obtain *sox5*^*wt or ml-3*^*-2A-venus*, *sox5* sequences were amplified from cDNA of WT or *ml-3* mutant by following primers and subsequently cloned into pGEM T-easy vector; WT form of *sox5*; 5’-GAATTCATGCTCACTGAGCCTGAGCTACCT-3’ (forward) and 5’-GTCGACTTGGGTGATGTGGTTCTCCTTGTC-3’ (reverse), *ml-3* form of *sox5*; 5’-GAATTCATGCTCACTGAGCCTGAGCTACCT-3’ (forward) and 5’-GTCGACTGGAGCTGATGGGGCCCAGCTTGG-3’ (reverse). Then, *sox5* fragment digested by EcoRI and SalI, and 2A-Venus fragment digested by SalI and XbaI from pBLSK-2A-venus that contains the 2A peptide sequence from *Thosea asigna* virus (TaV).

To obtain *loxp-dsred-loxp*-*sox5*^*wt or ml-3*^*-2A-venus* cassette, *β-actin pro*:*loxp-dsred-loxp-egfp* (kindly provided by Dr. Kinoshita) was first digested by SalI and SmaI and the *loxp-dsred-loxp* fragment inserted into PL451 digested by SalI and EcoRV. Next, to insert *sox5*^*wt or ml-3*^*-2A-venus* into the second loxp site of PL451*-loxp-dsred-loxp*, pCSII-*sox5*^*wt or ml-3*^*-2A-venus* was digested by EcoRI and NsiI and PL451*-loxp-dsred-loxp* was digested by EcoRI. The fragments produced were treated with KOD polymerase to form their end into blunt end, and then they were ligated. To make BAC construct, the sequence of *pax7a* exon 1 downstream of the start codon was replaced with the *loxp-dsred-loxp*-*sox5*^*wt or ml-3*^*-2A-venus* cassette to obtain *pax7a*:*loxp-dsred-loxp*-*sox5*^*wt or ml-3*^*-2A-venus* vectors. The BAC was modified as described previously [64]. The primers used in the construction were followings; 5’-TGAAGGCTCGATCAGTGTCCAGTTGGGTGTTTTTGCCTGGGTGGCTAGATCCTCGAGGTCGACATAACTT-3’ (left), 5’-TCGAACACAAGTCCAAAAAAGAAGCACTCCTTGTCCCCTTCCTTACCTTCGTACCTGACTGATGAAGTTC-3’ (right). Transgenic strains used are: Nagoya;*TgBAC(pax7a*:*loxp-dsred-loxp-sox5*^*wt*^*-2A-venus)* and Nagoya; or *sox5*^*ml-3/ml-3*^*;TgBAC(pax7a*:*loxp-dsred-loxp-sox5*^*wt*^*-2A-venus;pax7a*:*loxp-dsred-loxp-sox5*^*ml-3*^*-2A-venus)*.

*Tg(hsp70*:*cre)* fish was a gift from Dr. J. Wittbrodt (EMBL). Cre recombinase was induced with heat shock at 40°C for 40 min.

### Genotyping

To genotype the medaka *sox10a*^*E2del16*^ allele, *sox10b*^*N108S*^, *sox10b*^*E1del7*^ alleles, and zebrafish *sox5*^*E4del7*^ allele, we detected increased mobility of PCR amplicon in 12% polyacrylamide gel electrophoresis due to the deletion [65, 66]. The PCR primers used are: medaka *sox10a*^*E1ins10*^; 5’-GCATTAATCCCTGGTGGATCC-3’ (forward) and 5’-GGCTGAGGCGGTGAGAGTGAGT-3’ (reverse) and *sox10a*^*E2del16*^; 5’-CTCCCTCTAGGCTGCTGAACGAGA-3’ (forward) and 5’-GAGACCCTGCGCCCACATTGTGAT-3’ (reverse), medaka *sox10b*^*N108S*^; 5’-CTGTTCTTCCGCCAAATCCGACGA-3’ (forward), 5’-CCACACCTGTCGGGTCTGTCA-3’ (reverse), medaka *sox10b*^*E1del7*^; 5’-AATGTCCAGGGAGGAGCAGAGCCT-3’ (forward) and 5’-GTCGTCGGATTTGGCGGAAGAACA-3’ (reverse), zebrafish *sox5*; 5’-TGAGAGGCTGTTGTCTAAGGA-3’ (forward) and 5’-TAAGCTCAGAGGTCACATGAA-3’ (reverse). As for *sox10b*^*N108S*^ allele, the PCR products were treated with XmnI (NEB): Only WT allele is digested, which can be detected as increased mobility in 1% agarose gel electrophoresis.

### Whole mount in situ hybridization and plastic sectioning

Whole mount in situ hybridization and plastic sectioning were performed as previously described [18]. The antisense riboprobes were as follows: *dct* and *mitfa* of medaka [18] and *sox10* of zebrafish [30]; and *sox10a*, *sox10b* and *ltk* were synthesized from the plasmid described above using SP6 polymerase (Promega) after restriction enzyme digestion: *sox10a* and *sox10b*; EcoRI, *ltk*; XhoI (NEB). For double fluorescent in situ hybridization, the probes were labeled with digoxigenin or fluorescein, and signals were detected with anti-DIG or anti-Fluor POD-conjugated antibodies with using Tyramide Signal Amplification system (Invitrogen). For sectioning, the stained samples were embedded in Technovit 8100 (Heraeus Kulzer) and sectioned at 10μm thickness. Images of stained embryos were taken using Leica MZ APO with attached AxioCam camera (Zeiss) or Zeiss AXIOImager.M2 with attached Orca-frash 4.0 camera. Images of sections were taken using Zeiss AxioPlan2 with an AxioCam camera. Confocal images were obtained with a Zeiss LSM880 laser-scanning confocal microscope.

### Immunohistochemistry

The anti-medaka Sox5 rabbit antiserum was prepared by using DYASDNENHITQ synthetic peptide as an epitope of antigen (corresponding to the residues 674–685 of 685 amino acid protein, accession number EF577484). Embryos were fixed in 4% PFA/PBS at 4°C overnight. If necessary, samples were treated with 3% H_2_O_2_ and 0.5% KOH in PBS to remove residual melanin before blocking. A primary antibody was diluted 1:300 in 5% goat serum/PBS solution. Biotin-conjugated anti-rabbit IgG (VECTOR) was used as secondary antibody at a 1:500 dilution. Signals were detected by using VECTASTAIN ABC Elite kit (VECTOR) with diaminobenzidine (DAB, MUTO PURE CHEMICALS). For double immunohistochemistry, in combination with anti-Sox5 rabbit antiserum, anti-Pax3/7 mouse monoclonal antibody DP311 ([67], kindly provided by Dr. Nipam H Patel) was used as a primary antibody to detect Pax7 protein (1:200). Secondary antibodies used were Alexa Fluor 488 goat anti-rabbit and Alexa Fluor 568 goat anti-mouse (Molecular Probes, 1:500).

### Observation of pigment cells

In order to count melanocytes, embryos or larvae were treated with 2mg/ml epinephrine to aggregate melanin for 30 minutes. Auto-fluorescence of xanthophores was detected by UV light exposure through DAPI filter. In order to count adult pigment cells, fish were treated with 1μM melatonin for 30 min. The images were taken as described above.

### RT-PCR

Ten medaka or zebrafish embryos for each stage were used to collect total RNA with Sepazol (SIGMA). Total RNAs were treated with RQ1 DNase to prevent genomic DNA contamination. One μg of total RNA for each stage and species was used for synthesis of first-strand cDNA with the M-MLV Reverse Transcriptase (Promega) using Random hexamer primer (Promega). The resultant cDNA samples were subjected to semi-quantitative PCR amplification (30 cycles of 94°C for 30 sec, 60°C for 30 sec and 72°C for 1 min) and run in an agarose gel. The band of PCR products was evaluated in comparison with a housekeeping gene, *ef1α*. Primer information is listed in Supplementary S1 Table.

## Supporting information

S1 Fig *sox10a* mRNA co-expresses with *sox5* expression in neural crest. (A) *sox10a*. (B) *sox10b*. (A, B) 16 somite stage (44 hpf). (C-E) Double in situ hybridization for *sox5* (green) and *sox10a* (magenta) in WT medaka embryos at 18–19 somite stage (50 hpf). Lateral views dorsal to the top.
(A) *sox10a* is expressed in migrating neural crest cells on the medial pathway (black arrow) and premigratory neural crest cells on posterior trunk (black arrow heads). (B) *sox10b* expression is similar to *sox10a* expression.
(C-C”) Fluorescence images obtained by wide-field microscopy. (C) *sox5* mRNA is detected in dorsal neural tube and neural crest cells in trunk. (C’) *sox10a* mRNA is detected in neural crest cells. (C”) *sox5* mRNA and *sox10a* mRNA show partially overlapping expression and hence are co-localized in neural crest. (D, E) Higher magnification images obtained by confocal microscopy. (D-D”) Images on anterior trunk. Some neural crest cells on medial pathway express both *sox5* and *sox10a* (white arrows). (E-E”) Images focused on posterior trunk. Premigratory neural crest cells on dorsal trunk show co-expression of *sox5* and *sox10a* (white arrowheads).
Scale bars: (C, D, E) 200 μm.
(TIF)

S2 Fig Mutations of *sox10* and *sox5* genes in medaka and zebrafish. The wild type *sox10* genes encode a protein comprising an HMG box domain (red box) and a C-terminal transactivation domain (blue box). The *sox10a*^*E2del16*^ mutant allele has a 16-base deletion in exon 2, resulting in a truncated Sox10a protein lacking the C-terminal of HMG DNA binding domain and the transactivation domain (Sox10a^E2del16^). The *sox10a*^*E1ins10*^ allele has a 10-base nucleotide insertion in exon 1, which results in introduction of a premature stop codon and complete absence of both HMG and transactivation domains (Sox10a^E1ins10^).
Two *sox10b* mutant alleles, *sox10b*^*N108S*^ and *sox10b*^*F110L*^, each have a distinct single nucleotide mutation in exon 2, which results in an amino acid substitution of a highly conserved amino acid in the HMG domain of Sox10b (Sox10b^N108S^ and Sox10b^F110L^). The *sox10b*^*E1del7*^ mutant allele, which has a 7-base nucleotide deletion in exon 1, results in lack of most functional domains.
Zebrafish Sox10^t3^ protein also lacks both the HMG and the transactivation domains. The Sox10a^baz1^ protein has a single amino acid substitution V117M in the HMG domain (NB N-terminal region of zebrafish Sox10 has 5 extra amino acids compared to that of medaka Sox10b) [23, 30], hence V117 in zebrafish Sox10 corresponds to V112 in medaka Sox10b.
Medaka *sox5*^*ml-3*^ allele is a spontaneous mutation leading to skipping of exon 7, which introduces a premature stop codon and results in a truncated Sox5 protein (Sox5^ml-3^) lacking one and a part of the two coiled-coil domains, a Q-box and the HMG domain [18]. Zebrafish Sox5^E4del7^ protein lacks all the functional domains due to a 7-base nucleotide deletion in exon 4 and a subsequent premature stop codon.
Grey box represents de novo C-terminus due to the altered reading frame.
Amino acid sequences of HMG box in Sox10s from medaka, zebrafish and mouse are aligned. The amino acid substitutions in the mutants (N108S, F110L in yellow and V117M in purple) are colored.
(TIF)

S3 Fig Medaka *ltk* is expressed in neural crest and differentiating iridoblasts. (A-C) Lateral views. (A’, B’, C’) Dorsal views.
At 12-somite stage (12s, 41 hpf), *ltk* is expressed in the premigratory neural crest (arrows) and in vicinity of eye (A, A’). At 18-somite stage (18s, 50 hpf), *ltk* expression in trunk neural crest extends more posteriorly, and on the eye (arrow) shows a punctate pattern consistent with choroidal iridophores (B, B’). At 34-somite stage (34s, 74 hpf), some weak *ltk*-expression is seen in neural crest cells of the dorsal trunk (C, arrowheads). The lateral patches (LP) show persistent strong *ltk* signals (C’).
Scale bars: (A, B, C) 200 μm, (C’) 40 μm.
(TIF)

S4 Fig Interaction of Sox5 and Sox10 influences late development of melanocytes and iridophores. (A-R) 9 dpf. The genotypes are all as indicated in the photos. (A-H) Lateral views. Transmitted light. (I-R) Dorsal views. Reflected light.
(S-X) Quantitation of pigment cell numbers. WT, n = 19; *sox10b*^*-/-*^, n = 16; *sox10a*^*-/-*^, n = 36; *sox10a*^*-/-*^*;sox10b*^*+/-*^, n = 22; *sox5*^*-/-*^ n = 20; *sox10b*^*-/-*^*;sox5*^*-/-*^, n = 10; *sox10a*^*-/-*^*;sox5*^*-/-*^, n = 32; *sox10a*^*-/-*^*;sox10b*^*+/-*^*;sox5*^*-/-*^, n = 23. Bars show mean and error bar (s.d.). Comparison between the groups was performed by Kruskal-Wallis test with SDCF post hoc test. *, *p*<0.05.
Scale bar: (A) 250 μm; (I) 100 μm.
(TIF)

S5 Fig Immunostaining of wild type medaka embryos with anti-Sox5 and anti-Pax7 antibodies. (A-C) Dorsolateral views. 90 hpf. (A’-C’) Higher magnification of the boxed region in A.
(A) Sox5 signals (green) were detected in xanthoblasts/xanthophores mainly located on lateral trunk (and some on dorsal trunk). (B) Pax7 signals (magenta) was expressed in both xanthophore and leucophore lineages. (C) Sox5 expressing xanthoblasts/xanthophores co-express Pax7 (white arrows in A’-C’), whereas in the dorsal trunk, Pax7-positive/Sox5-negative leucoblasts/leucophores were seen (white arrowheads in B’-C’).
(TIF)

S6 Fig RT-PCR analysis of *sox* genes. The experiment was performed using total RNA from 2–4 cell and 18-somite (18-som) stage embryos of either medaka or zebrafish. All genes examined show maternal expression.
(TIF)

S7 Fig Zebrafish *sox5* is expressed in premigratory neural crest similarly to *sox10*. (A, C, E) *sox5* expression. (B, D, F) *sox10* expression. (A-F) 18 hpf. (A, B) Lateral views. (C, D) Dorsal views. (E, F) Transverse sections.
Strong signal of *sox5* expression is detected in the head, tail bud, notochord and somites (A, C). A transverse section of the trunk region indicates that *sox5* is expressed in the premigratory neural crest cells (E, arrow). (B, D, F) *sox10* expression overlaps with *sox5* expression in the premigratory neural crest cells (F, arrow).
Scale bar: (A) 200 μm, (E) 20 μm.
(TIF)

S8 Fig Zebrafish homozygous for the *baz1* allele of *sox10* show milder pigment cell phenotypes than those for *t3* allele. (A, D, G) WT. (B, E, H) *sox10*^*baz1*^ mutant (*baz1*). (C, F, I) *colourless/sox10*^*t3*^ mutant (*t3*). (A-I) 5 dpf. (A-C, G-I) Lateral views of trunk. (D-F) Dorsal views. (D’-F’) UV images. Lateral views of trunk.
WT zebrafish larva has four melanocyte stripes in the trunk (A). The *baz1* (B) and *t3* mutants (C) lack the stripes. In WT, xanthophores are widely distributed on dorsal surface of head (D). The *baz1* mutant has a few xanthophores on head (E) and trunk (E’). The *t3* mutant almost entirely lacks visible xanthophores (F, F’). Iridophores lie along the dorsal, ventral and yolk sac melanocyte stripes in WT (G). A few iridophores are found in the dorsal stripe and often in the lateral patches (B) in *baz1* mutants (H). The *t3* mutant almost completely lacks iridophores (I), but residual cells may be present in the lateral patches (C).
Scale bars: (A, D, G) 200 μm, (E’) 100 μm.
(TIF)

S9 Fig Misexpression of mutated Sox5 does not affect pigment cell development. Nagoya*(sox5*^*+/+*^*);TgBAC(pax7a*:*loxp-dsred-loxp-sox5*^*ml-3*^,*pax7a*:*loxp-dsred-loxp-sox5*^*WT*^*)* was crossed with *Tg(hsp70*:*cre)* to obtain larvae where heat shock induces misexpression of Sox5 WT (triangle) or mutant (square) protein in the xanthophore/leucophore progenitors. The cross also produced larvae having no *sox5* transgenes, which are indicated as *sox5*: (Tg -) as well as larvae having both WT and mutant *sox5* transgenes. Formation of leucophores (grey) and melanocytes (black) was not altered by the heat shock induction of Sox5 mutant protein while misexpression of Sox5 WT protein reduced leucophores but not melanophores in the siblings.
Data from the hatchlings not treated with heat shock are presented as heat shock (-) and data from those not having the transgene are as *sox5* (Tg -).
Bars show mean and error bar (s.d.). Comparison between with and without heat shock was performed by paired t-test.
(TIF)

S1 Table Overview of primer sequences for RT-PCR. Some primer sets have been described in the following articles: medaka *ef1α* [68], medaka *sox9a* [69], medaka *sox9b* [70], zebrafish *ef1α* [71].
(DOCX)
