# Supplementary figures and images for: Distinct interactions of Sox5 and Sox10 in fate specification of pigment cells in medaka and zebrafish
Source: PLoS Genet. 2018 Apr 5;14(4):e1007260. doi: 10.1371/journal.pgen.1007260 (PMC5886393; doi:10.1371/journal.pgen.1007260)

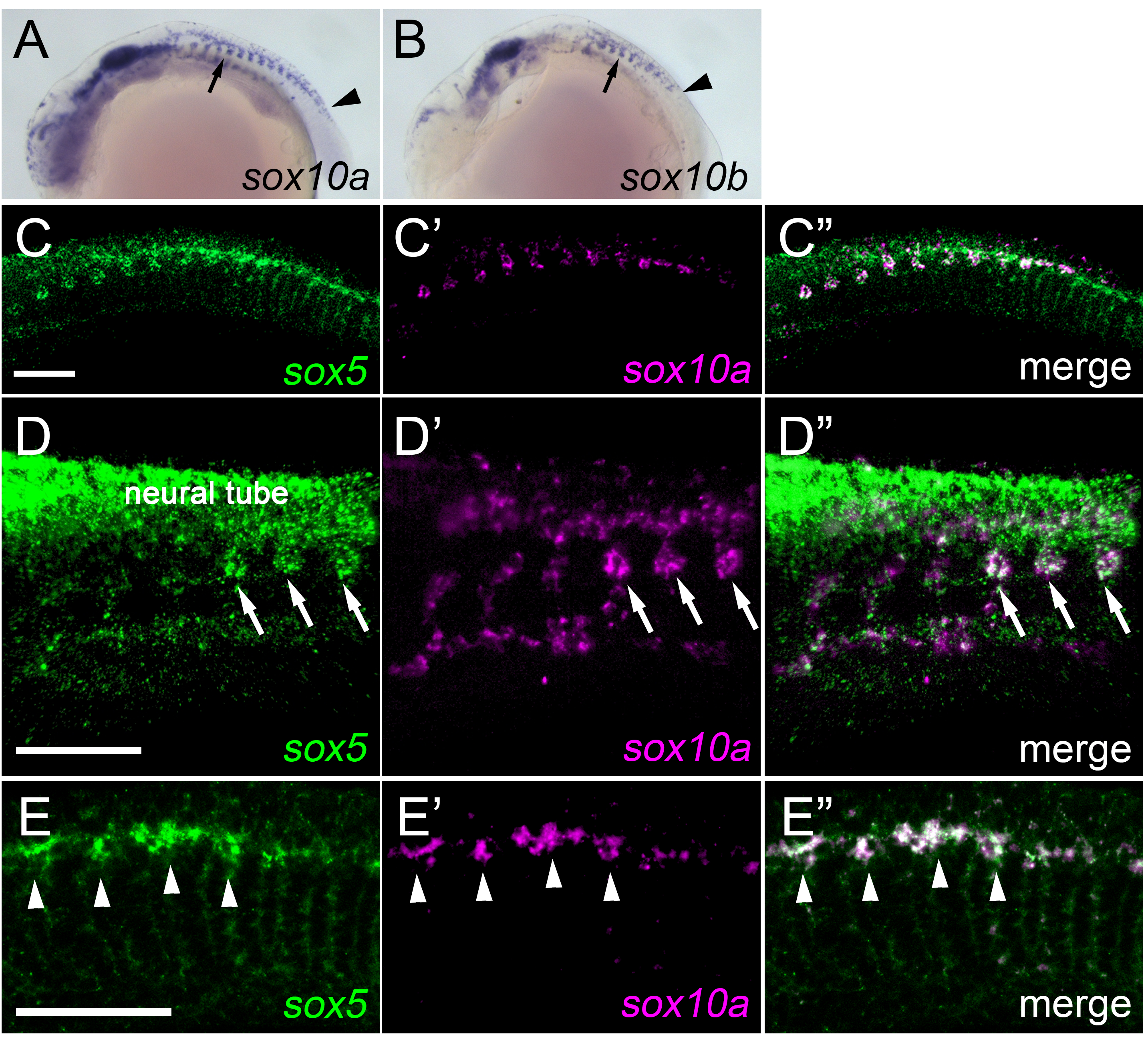

Supplement: S1 Fig — (A) sox10a. (B) sox10b. (A, B) 16 somite stage (44 hpf). (C-E) Double in situ hybridization for sox5 (green) and sox10a (magenta) in WT medaka embryos at 18–19 somite stage (50 hpf). Lateral views dorsal to the top. (A) sox10a is expressed in migrating neural crest cells on the medial pathway (black arrow) and premigratory neural crest cells on posterior trunk (black arrow heads). (B) sox10b expression is similar to sox10a expression. (C-C”) Fluorescence images obtained by wide-field microscopy. (C) sox5 mRNA is detected in dorsal neural tube and neural crest cells in trunk. (C’) sox10a mRNA is detected in neural crest cells. (C”) sox5 mRNA and sox10a mRNA show partially overlapping expression and hence are co-localized in neural crest. (D, E) Higher magnification images obtained by confocal microscopy. (D-D”) Images on anterior trunk. Some neural crest cells on medial pathway express both sox5 and sox10a (white arrows). (E-E”) Images focused on posterior trunk. Premigratory neural crest cells on dorsal trunk show co-expression of sox5 and sox10a (white arrowheads). Scale bars: (C, D, E) 200 μm. (TIF) [file pgen.1007260.s001.tif]

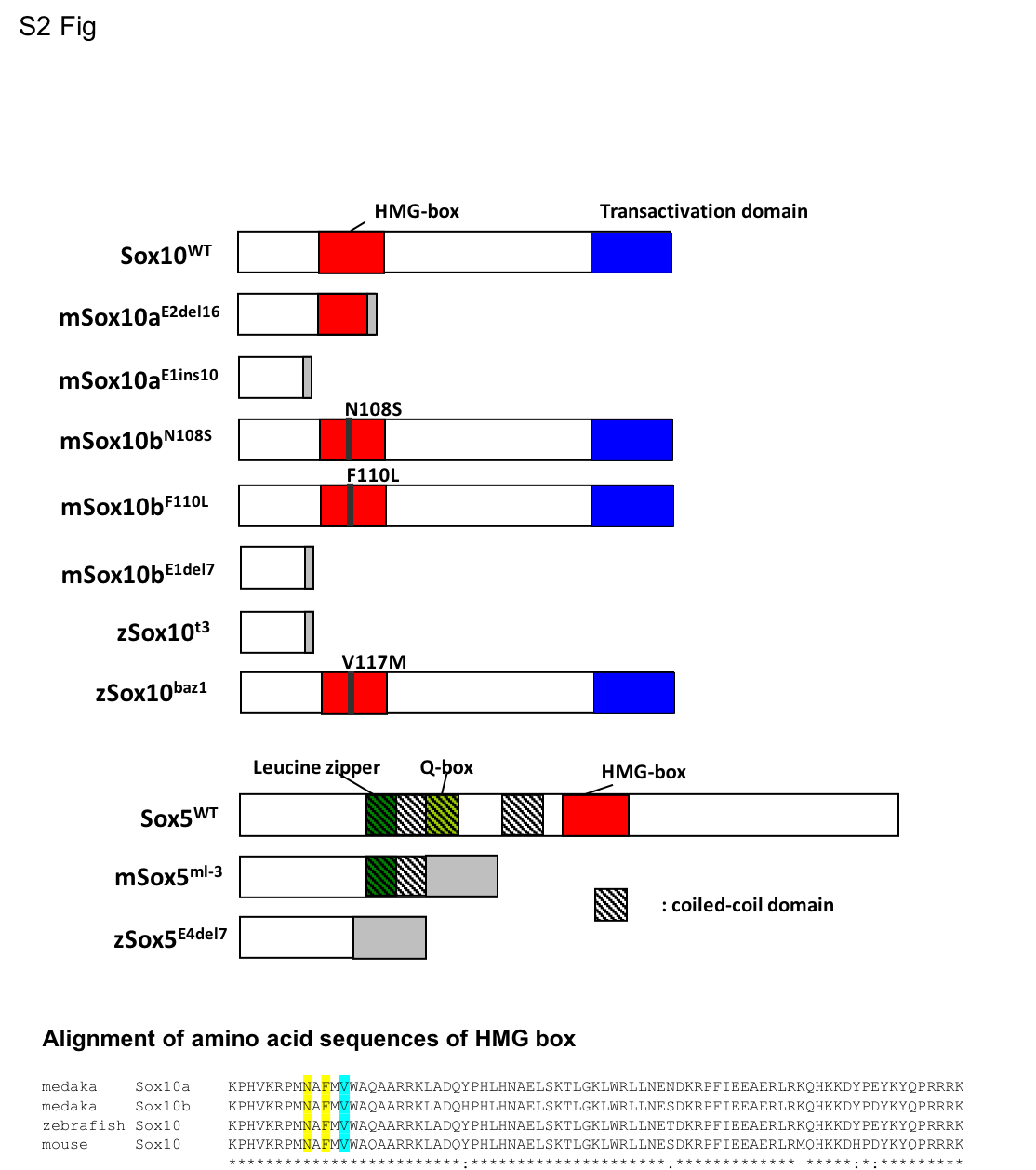

Supplement: S2 Fig — The wild type sox10 genes encode a protein comprising an HMG box domain (red box) and a C-terminal transactivation domain (blue box). The sox10aE2del16 mutant allele has a 16-base deletion in exon 2, resulting in a truncated Sox10a protein lacking the C-terminal of HMG DNA binding domain and the transactivation domain (Sox10aE2del16). The sox10aE1ins10 allele has a 10-base nucleotide insertion in exon 1, which results in introduction of a premature stop codon and complete absence of both HMG and transactivation domains (Sox10aE1ins10). Two sox10b mutant alleles, sox10bN108S and sox10bF110L, each have a distinct single nucleotide mutation in exon 2, which results in an amino acid substitution of a highly conserved amino acid in the HMG domain of Sox10b (Sox10bN108S and Sox10bF110L). The sox10bE1del7 mutant allele, which has a 7-base nucleotide deletion in exon 1, results in lack of most functional domains. Zebrafish Sox10t3 protein also lacks both the HMG and the transactivation domains. The Sox10abaz1 protein has a single amino acid substitution V117M in the HMG domain (NB N-terminal region of zebrafish Sox10 has 5 extra amino acids compared to that of medaka Sox10b) [23, 30], hence V117 in zebrafish Sox10 corresponds to V112 in medaka Sox10b. Medaka sox5ml-3 allele is a spontaneous mutation leading to skipping of exon 7, which introduces a premature stop codon and results in a truncated Sox5 protein (Sox5ml-3) lacking one and a part of the two coiled-coil domains, a Q-box and the HMG domain [18]. Zebrafish Sox5E4del7 protein lacks all the functional domains due to a 7-base nucleotide deletion in exon 4 and a subsequent premature stop codon. Grey box represents de novo C-terminus due to the altered reading frame. Amino acid sequences of HMG box in Sox10s from medaka, zebrafish and mouse are aligned. The amino acid substitutions in the mutants (N108S, F110L in yellow and V117M in purple) are colored. (TIF) [file pgen.1007260.s002.tif]

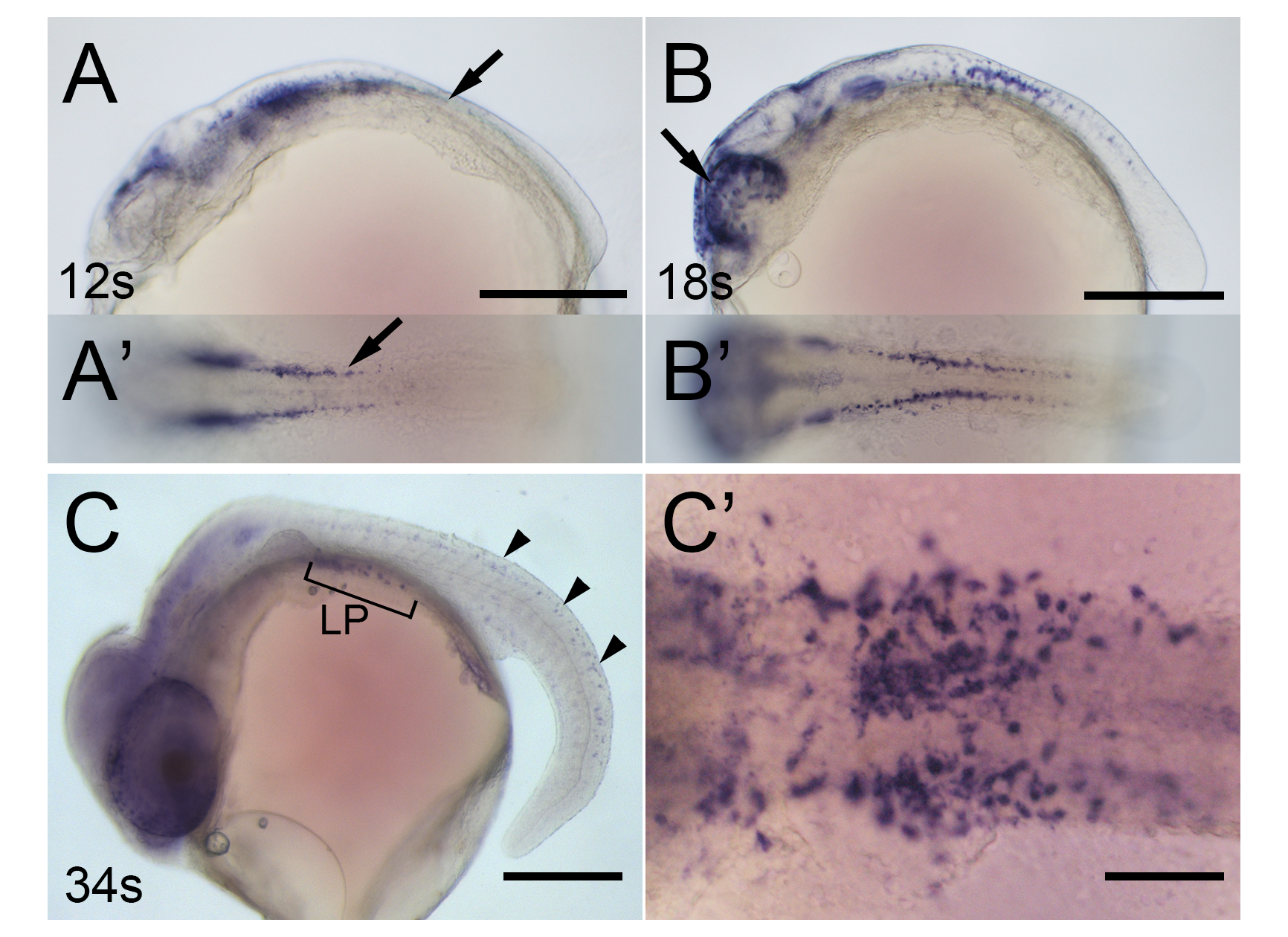

Supplement: S3 Fig — (A-C) Lateral views. (A’, B’, C’) Dorsal views. At 12-somite stage (12s, 41 hpf), ltk is expressed in the premigratory neural crest (arrows) and in vicinity of eye (A, A’). At 18-somite stage (18s, 50 hpf), ltk expression in trunk neural crest extends more posteriorly, and on the eye (arrow) shows a punctate pattern consistent with choroidal iridophores (B, B’). At 34-somite stage (34s, 74 hpf), some weak ltk-expression is seen in neural crest cells of the dorsal trunk (C, arrowheads). The lateral patches (LP) show persistent strong ltk signals (C’). Scale bars: (A, B, C) 200 μm, (C’) 40 μm. (TIF) [file pgen.1007260.s003.tif]

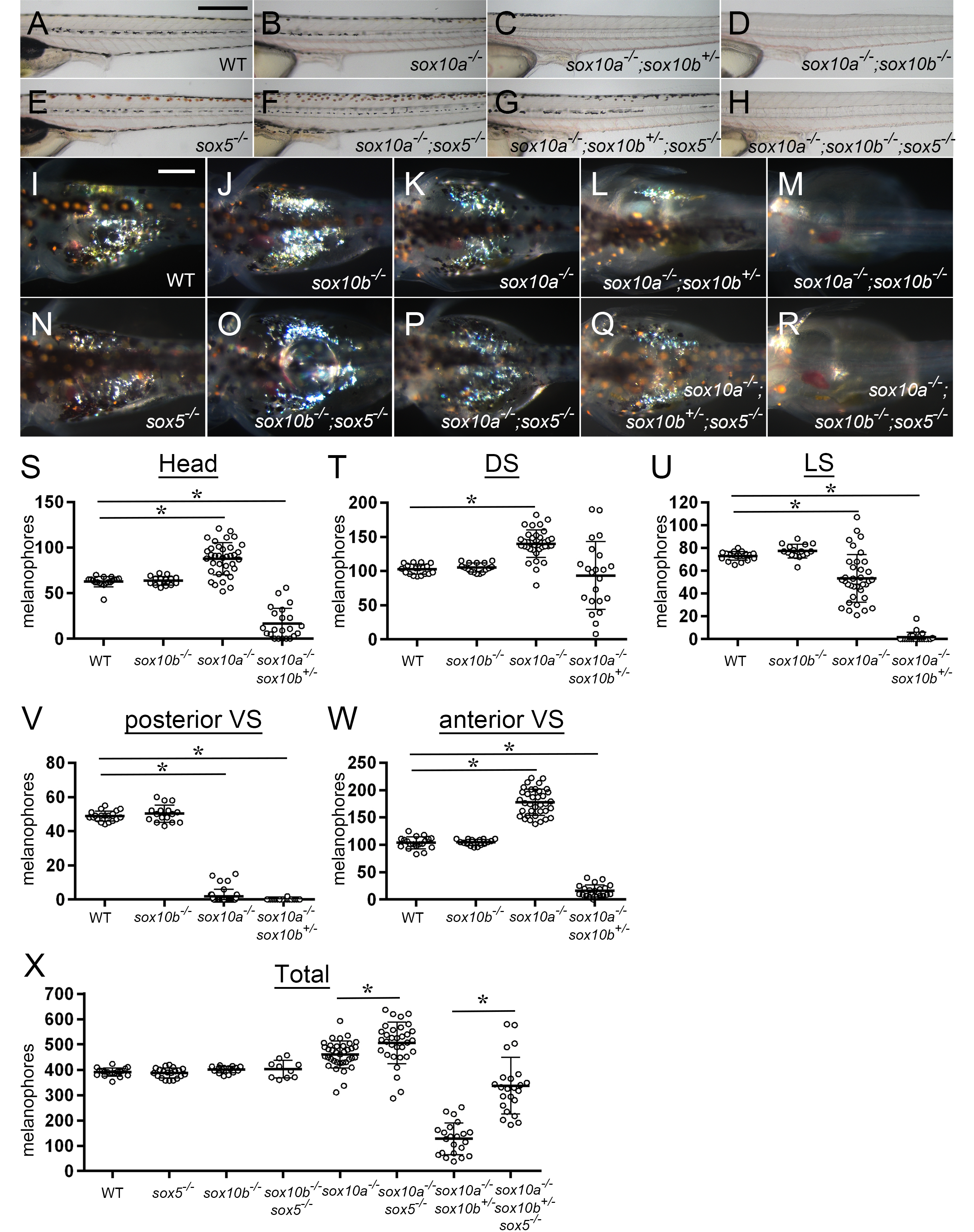

Supplement: S4 Fig — (A-R) 9 dpf. The genotypes are all as indicated in the photos. (A-H) Lateral views. Transmitted light. (I-R) Dorsal views. Reflected light. (S-X) Quantitation of pigment cell numbers. WT, n = 19; sox10b-/-, n = 16; sox10a-/-, n = 36; sox10a-/-;sox10b+/-, n = 22; sox5-/- n = 20; sox10b-/-;sox5-/-, n = 10; sox10a-/-;sox5-/-, n = 32; sox10a-/-;sox10b+/-;sox5-/-, n = 23. Bars show mean and error bar (s.d.). Comparison between the groups was performed by Kruskal-Wallis test with SDCF post hoc test. *, p<0.05. Scale bar: (A) 250 μm; (I) 100 μm. (TIF) [file pgen.1007260.s004.tif]

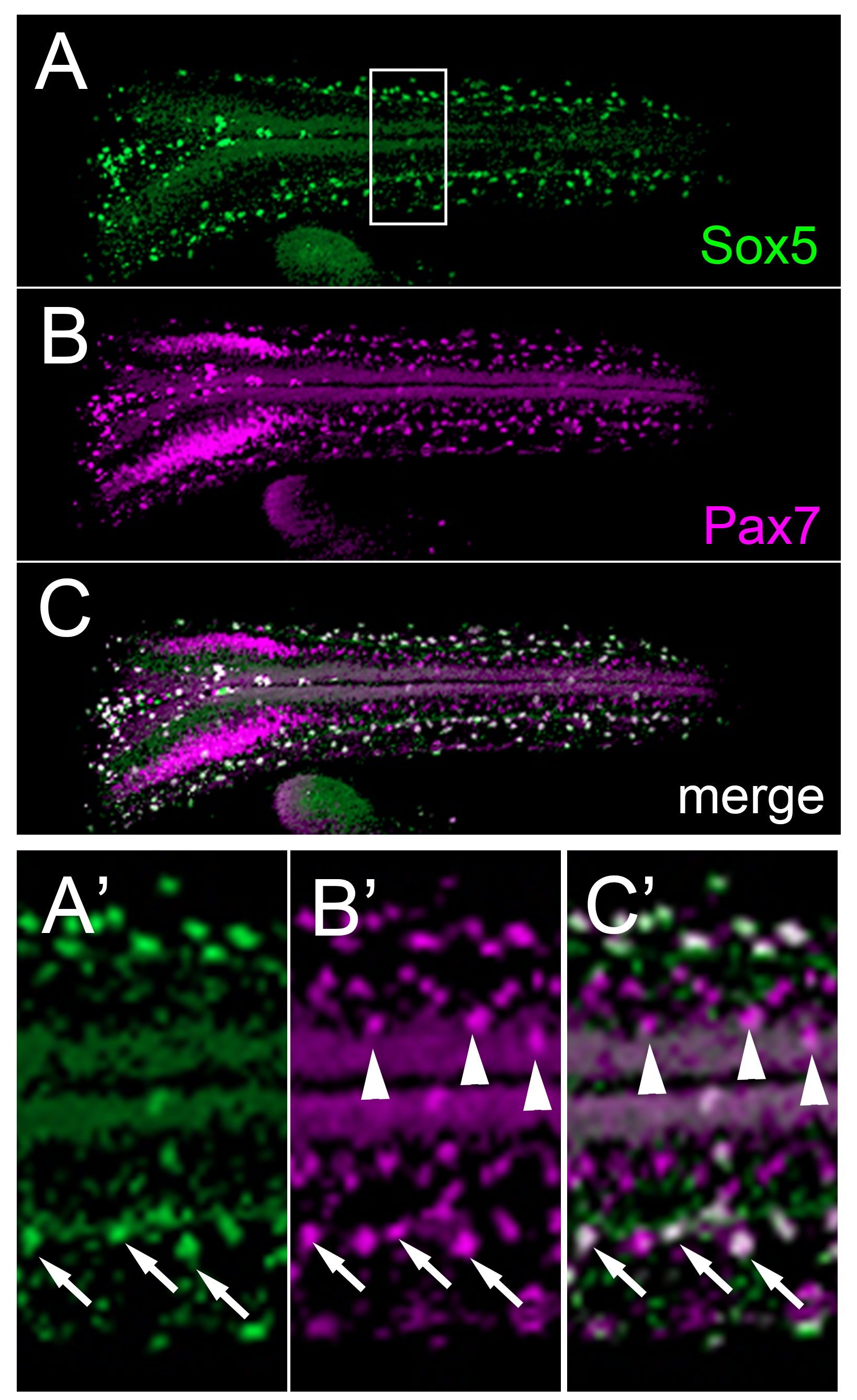

Supplement: S5 Fig — (A-C) Dorsolateral views. 90 hpf. (A’-C’) Higher magnification of the boxed region in A. (A) Sox5 signals (green) were detected in xanthoblasts/xanthophores mainly located on lateral trunk (and some on dorsal trunk). (B) Pax7 signals (magenta) was expressed in both xanthophore and leucophore lineages. (C) Sox5 expressing xanthoblasts/xanthophores co-express Pax7 (white arrows in A’-C’), whereas in the dorsal trunk, Pax7-positive/Sox5-negative leucoblasts/leucophores were seen (white arrowheads in B’-C’). (TIF) [file pgen.1007260.s005.tif]

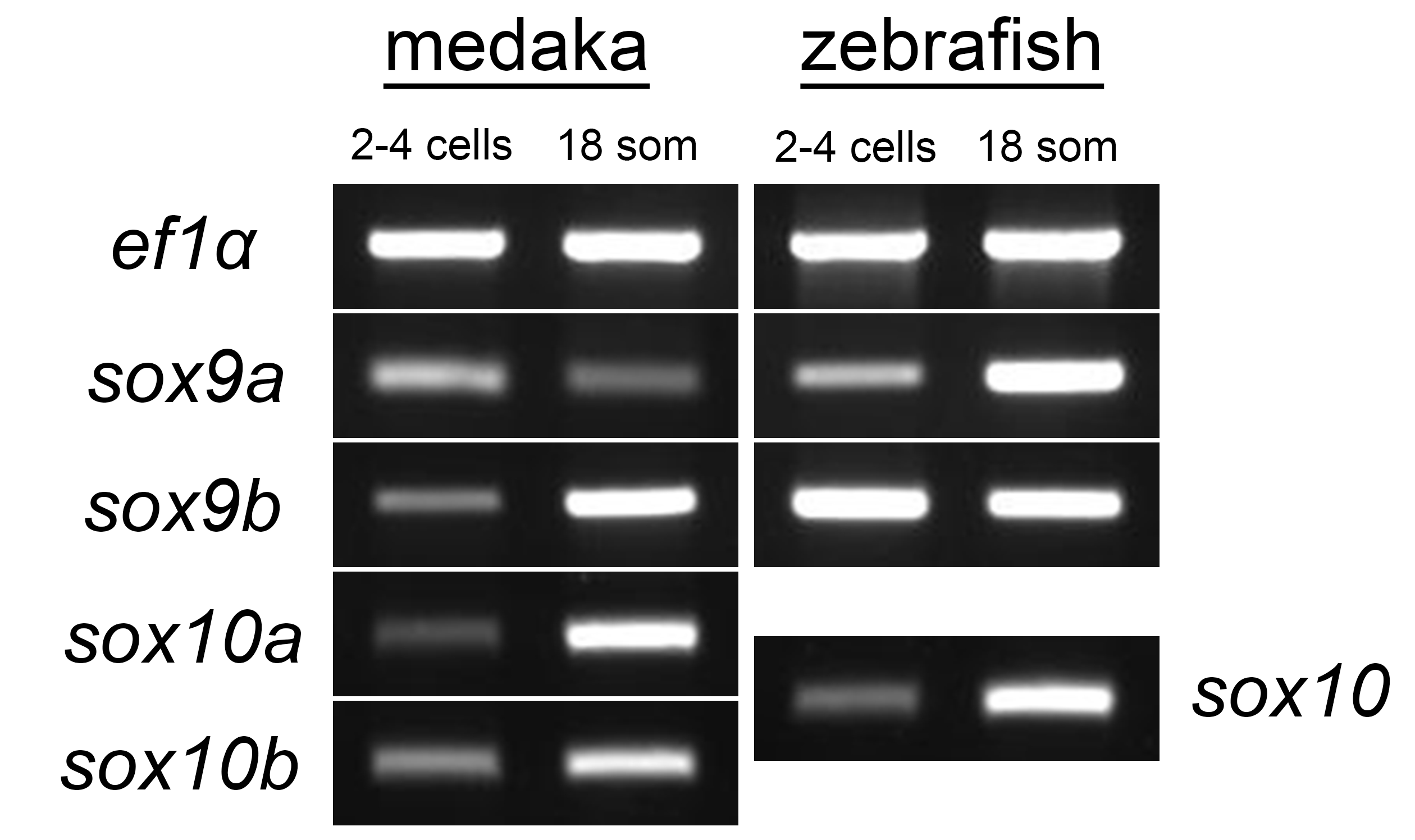

Supplement: S6 Fig — The experiment was performed using total RNA from 2–4 cell and 18-somite (18-som) stage embryos of either medaka or zebrafish. All genes examined show maternal expression. (TIF) [file pgen.1007260.s006.tif]

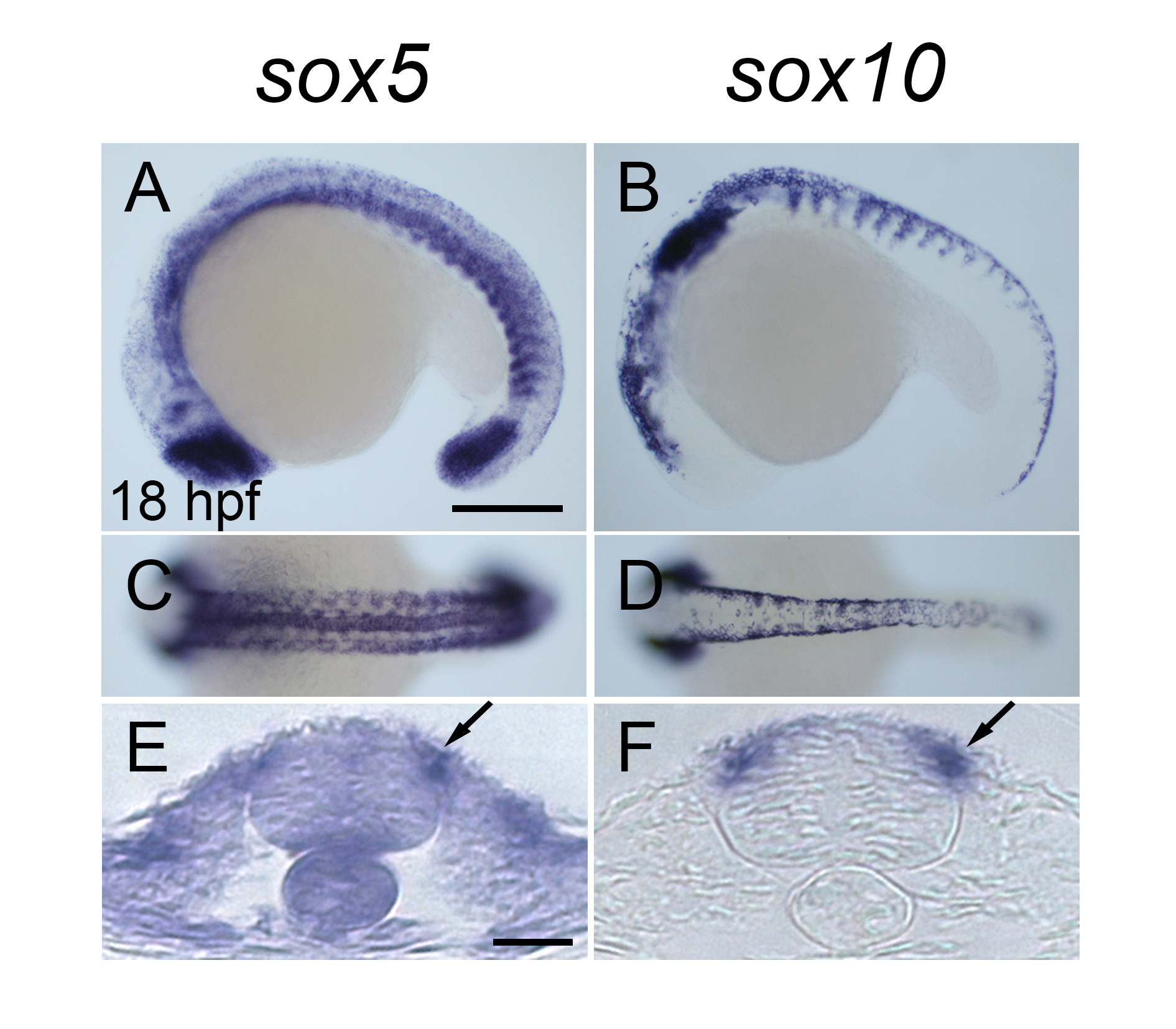

Supplement: S7 Fig — (A, C, E) sox5 expression. (B, D, F) sox10 expression. (A-F) 18 hpf. (A, B) Lateral views. (C, D) Dorsal views. (E, F) Transverse sections. Strong signal of sox5 expression is detected in the head, tail bud, notochord and somites (A, C). A transverse section of the trunk region indicates that sox5 is expressed in the premigratory neural crest cells (E, arrow). (B, D, F) sox10 expression overlaps with sox5 expression in the premigratory neural crest cells (F, arrow). Scale bar: (A) 200 μm, (E) 20 μm. (TIF) [file pgen.1007260.s007.tif]

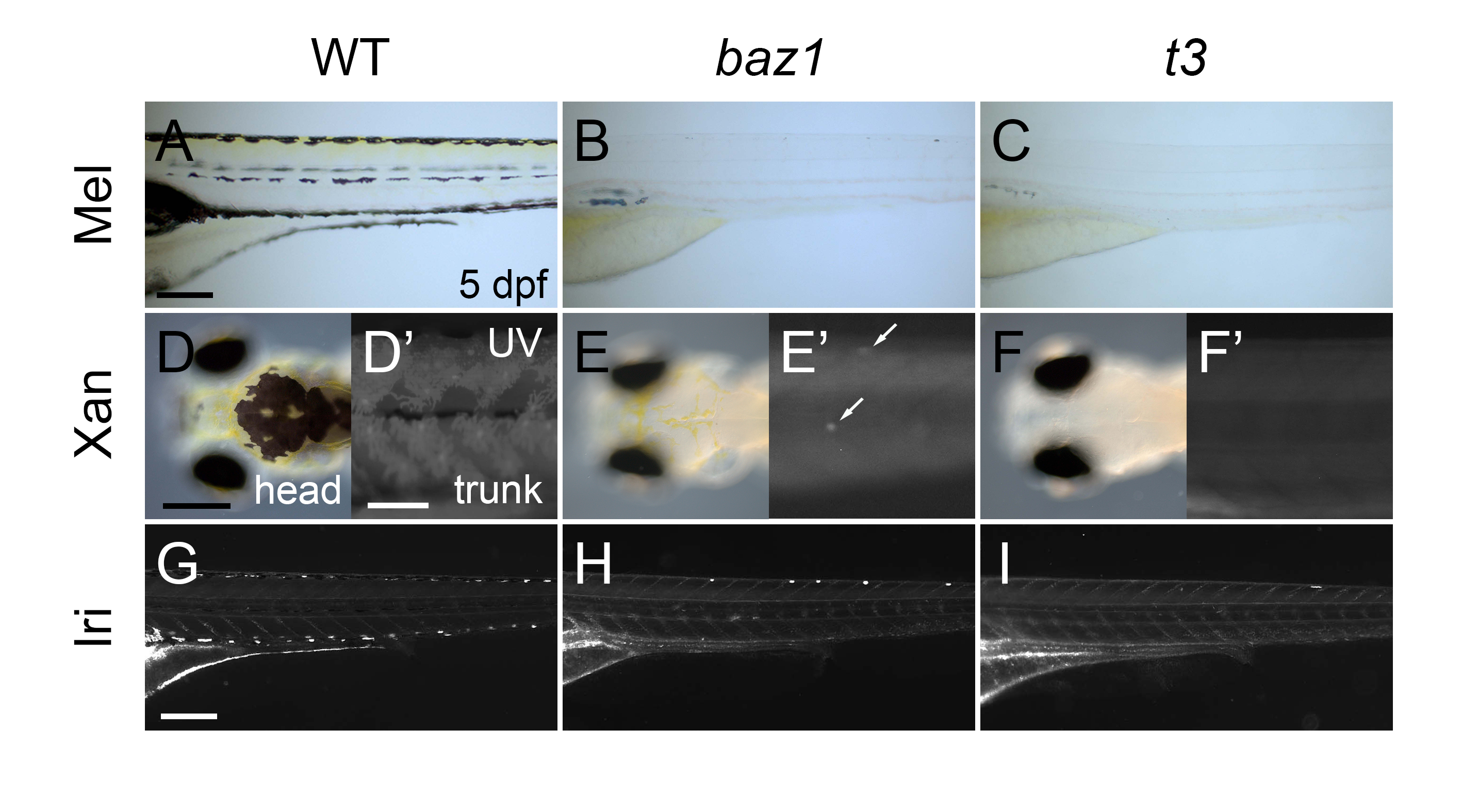

Supplement: S8 Fig — (A, D, G) WT. (B, E, H) sox10baz1 mutant (baz1). (C, F, I) colourless/sox10t3 mutant (t3). (A-I) 5 dpf. (A-C, G-I) Lateral views of trunk. (D-F) Dorsal views. (D’-F’) UV images. Lateral views of trunk. WT zebrafish larva has four melanocyte stripes in the trunk (A). The baz1 (B) and t3 mutants (C) lack the stripes. In WT, xanthophores are widely distributed on dorsal surface of head (D). The baz1 mutant has a few xanthophores on head (E) and trunk (E’). The t3 mutant almost entirely lacks visible xanthophores (F, F’). Iridophores lie along the dorsal, ventral and yolk sac melanocyte stripes in WT (G). A few iridophores are found in the dorsal stripe and often in the lateral patches (B) in baz1 mutants (H). The t3 mutant almost completely lacks iridophores (I), but residual cells may be present in the lateral patches (C). Scale bars: (A, D, G) 200 μm, (E’) 100 μm. (TIF) [file pgen.1007260.s008.tif]

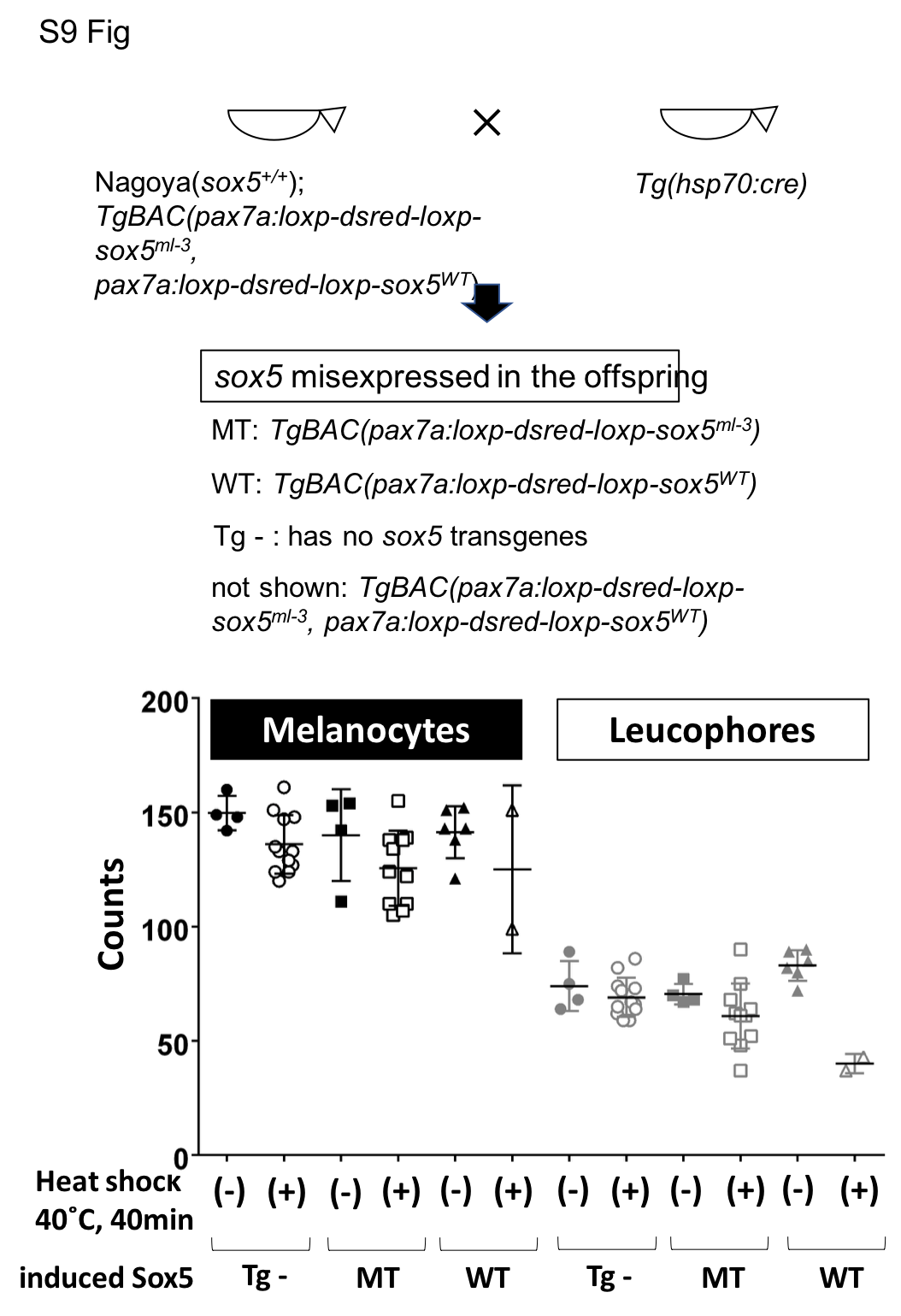

Supplement: S9 Fig — Nagoya(sox5+/+);TgBAC(pax7a:loxp-dsred-loxp-sox5ml-3,pax7a:loxp-dsred-loxp-sox5WT) was crossed with Tg(hsp70:cre) to obtain larvae where heat shock induces misexpression of Sox5 WT (triangle) or mutant (square) protein in the xanthophore/leucophore progenitors. The cross also produced larvae having no sox5 transgenes, which are indicated as sox5: (Tg -) as well as larvae having both WT and mutant sox5 transgenes. Formation of leucophores (grey) and melanocytes (black) was not altered by the heat shock induction of Sox5 mutant protein while misexpression of Sox5 WT protein reduced leucophores but not melanophores in the siblings. Data from the hatchlings not treated with heat shock are presented as heat shock (-) and data from those not having the transgene are as sox5 (Tg -). Bars show mean and error bar (s.d.). Comparison between with and without heat shock was performed by paired t-test. (TIF) [file pgen.1007260.s009.tif]
