# Supplementary material for: Distinct interactions of Sox5 and Sox10 in fate specification of pigment cells in medaka and zebrafish
Source: PLoS Genet. 2018 Apr 5;14(4):e1007260. doi: 10.1371/journal.pgen.1007260 (PMC5886393; doi:10.1371/journal.pgen.1007260)
Supplement: S1 Table — Some primer sets have been described in the following articles: medaka ef1α [68], medaka sox9a [69], medaka sox9b [70], zebrafish ef1α [71]. (DOCX) [file pgen.1007260.s010.docx]

S1 Table. Overview of primer sequences for RT-PCR

|  | Forward Primer | Reverse Primer |
| --- | --- | --- |
| medaka ef1α | CAGGACGTCTACAAAATCGG | AGCTCGTTGAACTTGCAGGCG |
| medaka sox9a | ATCTTCAAGGCTCTGCAGCA | ACGTCGAACGTCTCAATGTG |
| medaka sox9b | CTCCAGGAGAACATTCAGGT | CAAAGAATGCTGCTGTTTGG |
| medaka sox10a | GACGATCGGTTTCCCATTGGGAT | GCCCACATTGTGATCCAGGTGTA |
| medaka sox10b | GACGATCGGTTTCCCATTGGGAT | GTCCGTCAGCCTCACTTCCAGAT |
| zebrafish ef1α | AGCCTGGTATGGTTGTGACCTTCG | CCAAGTTGTTTTCCTTTCCTGCG |
| zebrafish sox9a | GAACGGCCAGAGCGAATCTGAAGA | CCACGTCCTGGAAGTTGATGCTGA |
| zebrafish sox9b | CAAGAGACCCATGAACGCGTTTAT | ATCTGCGTCTGCTCTCCATCTTCA |
| zebrafish sox10 | TGAACTCGGGCAGCAAGAGCAAA | GTAATGCGATTGGCTGTGGCTGA |
